# The Transcriptome of *Nacobbus aberrans* Reveals Insights into the Evolution of Sedentary Endoparasitism in Plant-Parasitic Nematodes

**DOI:** 10.1093/gbe/evu171

**Published:** 2014-08-13

**Authors:** Sebastian Eves-van den Akker, Catherine J. Lilley, Etienne G. J. Danchin, Corinne Rancurel, Peter J. A. Cock, Peter E. Urwin, John T. Jones

**Affiliations:** ^1^Centre for Plant Sciences, University of Leeds, United Kingdom; ^2^Cell and Molecular Sciences Group, Dundee Effector Consortium, James Hutton Institute, Dundee, United Kingdom; ^3^Centre National de la Recherche Scientifique, INRA Institut National de la Recherche Agronomique, UMR 1355, Université de Nice-Sophia Antipolis, Sophia-Antipolis, France; ^4^Information and Computational Sciences Group, Dundee Effector Consortium, James Hutton Institute, Dundee, United Kingdom

**Keywords:** plant-parasitic nematode, RNAseq, lateral gene transfer, host mimics, phylogenetics, effectors

## Abstract

Within the phylum Nematoda, plant-parasitism is hypothesized to have arisen independently on at least four occasions. The most economically damaging plant-parasitic nematode species, and consequently the most widely studied, are those that feed as they migrate destructively through host roots causing necrotic lesions (migratory endoparasites) and those that modify host root tissue to create a nutrient sink from which they feed (sedentary endoparasites). The false root-knot nematode *Nacobbus aberrans* is the only known species to have both migratory endoparasitic and sedentary endoparasitic stages within its life cycle. Moreover, its sedentary stage appears to have characteristics of both the root-knot and the cyst nematodes. We present the first large-scale genetic resource of any false-root knot nematode species. We use RNAseq to describe relative abundance changes in all expressed genes across the life cycle to provide interesting insights into the biology of this nematode as it transitions between modes of parasitism. A multigene phylogenetic analysis of *N. aberrans* with respect to plant-parasitic nematodes of all groups confirms its proximity to both cyst and root-knot nematodes. We present a transcriptome-wide analysis of both lateral gene transfer events and the effector complement. Comparing parasitism genes of typical root-knot and cyst nematodes to those of *N. aberrans* has revealed interesting similarities. Importantly, genes that were believed to be either cyst nematode, or root-knot nematode, “specific” have both been identified in *N. aberrans*. Our results provide insights into the characteristics of a common ancestor and the evolution of sedentary endoparasitism of plants by nematodes.

## Introduction

Plant-parasitic nematodes (PPN) cause damage to almost all crops grown and losses to global agriculture attributed to PPNs are estimated at £75 billion per year (Nicol et al. 2011). The ability to parasitise plants is hypothesized to have arisen independently on at least four occasions in the Phylum Nematoda. Phylum-wide analysis of a single Small Subunit ribosomal DNA (SSU rDNA) has produced the most extensive phylogeny of nematodes to date. This phylogeny splits nematodes into 12 Clades, with plant parasites present in four of these Clades (van Megen et al. 2009). Clades 1 and 2 contain migratory ectoparasitic nematodes that remain outside the host for the duration of the life cycle. Clade 10, which is composed mainly of fungivorous nematodes, includes two plant-parasitic species of *Bursaphelenchus* (e.g., *Bursaphelenchus xylophilus*) and plant-parasitic (foliar) *Aphelenchoides* species (e.g., *Aphelenchoides fragariae*). Clade 12 contains insect parasites, vertebrate parasites, and the majority of the known plant-parasitic species, including those that are the most economically important.

PPN that employ several different feeding strategies are found within Clade 12. Migratory endoparasites cause considerable damage as they migrate through and feed from disrupted cells. These nematodes have no biotrophic stage. In addition, Clade 12 includes two groups of biotrophic sedentary endoparasitic nematodes that are thought to have evolved biotrophy independently. The best known examples from these two groups are the cyst nematodes and the root-knot nematodes, which are the most economically damaging species (Jones et al. 2013). The “false root-knot nematode” *Nacobbus aberrans* is highly unusual as it is the only known nematode that has both a migratory endoparasitic stage and a sedentary (biotrophic) stage within its life cycle.

**Fig. 1.—:**
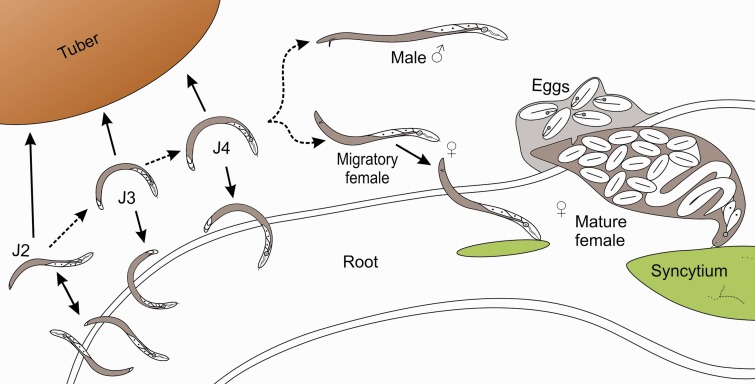
The life cycle of *Nacobbus* species on potato. Dashed arrows represent transitions through the life cycle, whereas solid arrows represent movement of the nematode. J2s hatch from eggs in the soil and locate plant roots. J2s migrate destructively (i.e., intracellularly) through roots. J2s moult without feeding, either in roots or in the soil, into the J3. This stage, and the subsequent J4, can enter or leave roots and also migrate destructively and in addition cause lesions and necrosis. The J3 and J4 are the dauer (survival) stages and are able to tolerate adverse environmental conditions such as low humidity. After the final moult the vermiform females migrate to healthy root tissue, where they establish a permanent plant-derived, multinucleate feeding site known as a syncytium that is formed inside a root gall. Females feed from the syncytium for several weeks as their body swells and eggs are produced. Eggs are deposited in a gelatinous matrix to form egg masses in the soil.

The complete life cycle of *N. aberrans* on a potato host is summarized in figure 1. Second-stage juveniles (J2s) hatch from eggs in the soil and locate plant roots. The J2s use coordinated movements of their needle-like stylet to puncture cells and then migrate destructively through them (intracellular migration) (Manzanilla-López et al. 2002). J2s moult without feeding, either in roots or in the soil, into the third-stage juvenile (J3). This stage, and the subsequent J4, can enter or leave roots, migrates destructively and also causes lesions and necrosis. It is thought that the migratory J3 and J4 stages feed, although mixed reports on this topic exist in the literature (Manzanilla-López et al. 2002; Doncaster 2012). The J3 and J4 are survival stages (equivalent to the *Caenorhabditis elegans* dauer stage) and are able to tolerate adverse environmental conditions such as low humidity (Anthoine et al. 2006). These stages can remain viable in potato roots/tubers for many months, causing very few visible symptoms, making them easily traded and a concern for quarantine legislation. After the final moult the vermiform females migrate to/into healthy root tissue, where they establish a permanent plant-derived multinucleate feeding site known as a syncytium, within a root gall (Jones and Payne 1977; Vovlas et al. 2007). An unusual feature of the complex life cycle of *N. aberrans* is that the complete development from J2 to female can occur either inside or outside the host root (Manzanilla-López et al. 2002). Females feed from the syncytium for several weeks as their body swells and egg production occurs. Eggs are deposited in a gelatinous matrix to form egg masses in the soil. Reproduction in *N. aberrans* is probably sexual, although there are some suggestions that parthenogenesis may occur (Manzanilla-López et al. 2002).

*Nacobbus aberrans* is thought to originate in South America, from where it has subsequently spread to the rest of the Americas, Europe, and Asia (Manzanilla-López 2010; Jones et al. 2013). It has a broad host range encompassing 84 known plant species across 18 families including crops such as potato, tomato, beans and sugar beet, and many common weeds (Brodie et al. 1993; Manzanilla-López et al. 2002; Manzanilla-López 2010). *Nacobbus aberrans* causes yield losses that vary with temperature, but can be up to 61% on potato (Franco 1994; Manzanilla-López et al. 2002). Due to a combination of the wide distribution, broad host range, potential yield losses, and peculiar dormant parasitic stage, *N. aberrans* is recognized as an EPPO A1 rated quarantine pest in Europe (Smith et al. 1992) and also has quarantine status in the United States (Lehman 2004). These factors led to *N. aberrans* being voted as one of the top ten nematodes in molecular plant pathology in a recent survey and review (Jones et al. 2013). *Nacobbus* spp, commonly referred to as false root-knot nematodes, are thought to be more closely related to the root-knot nematodes than the cyst nematodes (Holterman et al. 2006, 2009; van Megen et al. 2009). However, the syncytial feeding sites that they produce closely resemble those induced by cyst nematodes and have little in common with the giant cells induced by root-knot nematodes (Jones and Payne 1977). The syncytium, which is the sole source of nutrients for the adult female nematode over a period of several weeks, is formed by modifying an initial host cell to become more metabolically active. This initial cell then undergoes local cell wall dissolution and its protoplast fuses with those of the neighbouring cells which thus become incorporated into the syncytium. This process of syncytial formation is very similar to that seen in syncytia induced by cyst nematodes. Conversely, the syncytium is formed inside a gall that is phenotypically similar to that of the root-knot nematodes. In addition, the egg masses produced within/from this gall resemble those of the reniform and root-knot nematodes.

As for other plant pathogens, the interactions of PPN with their hosts are mediated by the activity of effectors. Effectors can be defined as proteins (or other factors) secreted by a parasite into a host in order to manipulate the host to the advantage of the parasite. Effectors clearly play a key role in several aspects of the biology of *N. aberrans.* Effectors that suppress host defences are likely to be produced as it remains undetected while feeding. As in other biotrophic PPN, effectors need to be produced that induce and maintain the feeding site. Various effectors from other nematode species have been linked to suppression of host defences and induction of the feeding site (Haegeman et al. 2012; Hewezi and Baum 2013). For example, a secreted SPRY domain-containing protein from the Clade 12 cyst nematode *Globodera rostochiensis* is able to supress CC-NB-LRR-mediated disease resistance responses (Postma et al. 2012). In addition, PPN have evolved short proteins/peptides that mimic endogenous plant peptide hormones. The CLAVATA-like (CLE peptides) of the cyst nematode *Heterodera schachtii* are apparently secreted into syncytia and subsequently transported to the apoplasm by plant mechanisms (Replogle et al. 2011). Although the precise role of the nematode peptides in parasitism is unclear, they appear to be functional in planta. A *H. glycines* CLE peptide can complement an *Arabidopsis thaliana* CLE mutant (Wang et al. 2005) and the overexpression of a nematode-derived CLE peptide in *A. thaliana* results in a “wuschel-like” phenotype similar to that caused by over expression of endogenous CLE peptides (Wang et al. 2011). A variety of genes encoding cell wall degrading enzymes have been identified from several PPN. These genes are highly similar to bacterial sequences, and absent from most other animals and are therefore likely to have been acquired by lateral gene transfer (LGT) (Danchin et al. 2010; Haegeman, Jones, et al. 2011).

In spite of the economic importance and highly unusual life cycle of *N. aberrans,* little is known about the molecular basis of host-parasite interactions in this species, and no resources for molecular studies are available. To address this, we have generated a “reference” transcriptome using RNAseq from three life stages of *N. aberrans.* We have subsequently analyzed changes in gene expression occurring across the life cycle, as it transitions between modes of parasitism. This analysis, including comparisons with other nematode groups, has allowed identification of putative effectors from *N. aberrans.* The data generated provide an insight into both the phylogenetic position of *N. aberrans* relative to other PPN and the evolution of sedentary endoparasitism by nematodes.

## Materials and Methods

### Biological Material

An isolate of *N. aberrans* was collected from Puno, Peru in 2001 and cultivated on tomato (*Solanum lycopersicum* var. Ailsa Craig) grown in compost in a greenhouse at 25 °C with a 16/8 h day/night cycle. Plants were not watered for 2–3 days before nematode extraction. Cleaned roots were cut into 2-cm sections and macerated using a bench top blender. Released eggs collected between 25- and 63-μm sieves were subjected to sucrose (40%) centrifugation at 4,000 × *g* for 4 min, washed using a 25-μm sieve, and placed in a hatching jar on top of a 25-μm mesh. Hatched nematodes were able to move through the mesh and were collected daily and flash frozen in liquid nitrogen. Only J2 nematodes collected between 4 and 7 days after initiation of hatching were used for RNA extraction. Mixed stage migratory nematodes (J3/J4/female), from this point onwards referred to as Mig or migratory, were collected using a similar blending procedure, but were present between 63- and 100-μm mesh sieves, sucrose centrifuged, individually separated from root debris by pipetting, and flash frozen in liquid nitrogen.

For collection of adult sedentary female nematodes, referred to as Sed, heavily infected cleaned root tissue was cut into 2-cm sections, and nematodes gently separated from galls by incubating with shaking (60 rpm) in 10 mg/ml Macerozyme (Duchefa Biochemie) at 30 °C for 5 h. Macerated tissue was sucrose centrifuged as above and females were collected between 100- and 500-μm sieves, manually separated from remaining debris, and flash frozen in liquid nitrogen. Three independent biological replicates were collected for each life stage and stored at −80 °C before RNA extraction.

### RNA Extraction and Sequencing

RNA extractions were carried out using RNeasy Spin columns (Qiagen) following the manufacturer’s instructions for animal tissues using the optional on-column DNase I digestion (Qiagen). RNA quality was assessed using a Nanodrop spectrophotometer (NanoDrop products) and a Bioanalyser (Agilent Technologies). RNA samples with RNA integrity number values from 8 to 10, absorbance ratios of 260:280 nm greater than 2 and 260:230 nm between 2 and 2.2 were used for sequencing using the service provided by The Genome Analysis Centre (TGAC, Norwich, UK). In brief, libraries were constructed using the PerkinElmer Sciclone with the TruSeq RNA protocol (Illumina). mRNA was separated from 1 μg of total RNA by poly-A pull down using biotin beads. mRNA was fragmented, cDNA synthesized, and overhanging ends repaired to create blunt ended DNA. 3′-A overhangs were added using *Taq* DNA polymerase, and ligated to corresponding 3′-T overhangs present on adapter sequences. Unligated adapters were removed using size selection XP beads (Beckman Coulter). The nine libraries, each prepared with different barcoded adapters, were normalized and pooled, spiked with 1% PhiX control v3, and sequenced on a single lane of Illumina HiSeq2000 using 100 cycles for each paired-end read.

### Assembly, Quality Control, and Differential Expression

A two-step procedure was used to analyse the RNAseq data. First, all data were pooled and used to generate a de novo whole transcriptome assembly. RNAseq reads from each individual life stage were subsequently mapped back to this assembly for further gene expression analysis.

Raw reads were trimmed of overrepresented adapter sequences and low-quality bases using Trimmomatic (Bolger et al. 2014). Two levels of trimming stringency were used; minimum phred quality scores of 22 and 28 were used for mapping and assembly, respectively. Overlapping pairs of high-quality reads were assembled into transcripts using Trinity (Grabherr et al. 2011) with default parameters.

Redundancy and misassembly among transcripts were removed using the following pipeline. CD-HIT-EST (http://weizhong-lab.ucsd.edu/cd-hit/, last accessed August 22, 2014) removed transcripts wholly contained within other transcripts at 99% identity. Postassembly transchimera removal was carried out by comparing all transcripts to a custom BLAST (Boratyn et al. 2013) database of all Nembase (http://www.nematodes.org/nembase4/, last accessed August 22, 2014) expressed sequence tag (EST) sequences. Transcripts were cut if to two different ESTs mapped to the same transcript in opposite reading frames, using a custom python script (Yang and Smith 2013). CD-HIT-EST at 99% was then repeated. All reads were mapped back to the transcriptome assembly using Bowtie2 (Trapnell et al. 2013), and the highest expressed isoform of each component was selected (Trinity-pickH) (Grabherr et al. 2011). All overlapping isoforms were “assembled” using Cap3 (−o 200 -p 99) (Huang and Madan 1999), and from the “nonassembled” transcripts all remaining isoforms present in the pickH list were kept. CD-HIT-EST at 97% identity was repeated to create a set of reference transcripts different enough to warrant independent analysis. Postassembly contamination removal was carried out using a BLAST-based approach as described in supplementary section S1, Supplementary Material online.

Trimmed RNA reads from each sample were mapped back to this reference transcriptome using Bowtie2. Trinity wrapper scripts for RSEM (Li and Dewey 2011) were used to generate read counts that were normalized by EdgeR (TMM) (Robinson et al. 2010) and calculate differentially expressed transcripts (*P* < 0.001, minimum fold change of 4). Differentially expressed transcripts were clustered based on their expression profile as 20% tree height, and then manually assigned to ten super clusters.

### Prediction of Proteins, Conserved Domains, and Gene Ontology Annotation

Protein-coding sequences were predicted from the assembled transcriptome using default parameters of TransDecoder (Haas et al. 2013). Pfam-A domains (Bateman et al. 2004) were identified from the predicted protein sets of *N. aberrans*, the root-knot nematodes *Meloidogyne incognita* (Abad et al. 2008) and *M. hapla* (Opperman et al. 2008), the cyst nematode *G. pallida* (Cotton et al. 2014), and the free-living nematode *C. elegans* (Consortium 1998) using the Pfam-Scan perl script based on HMMER (Eddy 2011). Only Pfam-A curated domains were searched for comparisons between species. Gene ontology (GO) terms were assigned to predicted proteins using the BLAST2GO software (Conesa et al. 2005).

### Detection of LGTs and Putative Contamination

To detect candidate LGTs, we calculated an Alien Index (AI) analysis as described in (Gladyshev et al. 2008; Flot et al. 2013). Briefly, all the *N. aberrans* predicted proteins and peptides were compared against the NCBI nonredundant library using BLASTp with an *e* value threshold of 1e^−3^ and no SEG filtering. BLAST hits were parsed to retrieve associated taxonomic information. An AI was calculated for each *N. aberrans* protein returning at least one hit in either a metazoan or nonmetazoan species using the following formula:

$$\begin{array}{l} \text{AI}=\log(\text{best metazoan }e\text{\_value+}e^{-200})\\ -\log(\text{best non\_metazoan }e\text{\_value}+e^{-200}) \end{array}$$

When no metazoan or nonmetazoan significant BLAST hit was found, an *e* value of 1 was automatically assigned. BLAST hits to Tylenchida nematodes were ignored for the calculation of AI to allow detection of LGT events in an ancestor of *N. aberrans* that are shared with other clade 12 PPN. No AI value could be calculated for *N. aberrans* proteins returning no significant hit at all in the nonredundant protein database. An AI >0 indicates a better hit to a nonmetazoan species than to a metazoan species. An AI >30 corresponds to a difference of magnitude e^10^ between the best nonmetazoan and best metazoan *e* values and is estimated to be indicative of a LGT event (Flot et al. 2013). All *N. aberrans* proteins that returned an AI >0 and that aligned with ≥70% identity to a nonmetazoan protein were considered as possible contaminants and were discarded from the analysis.

### Multigene Phylogenetic Analysis of Sedentary PPN

CEGMA (Parra et al. 2007) was used to predict core eukaryotic genes (CEGs) from the genomes of *G. pallida, G. rostochiensis, M. incognita, M. hapla, M. javanica, M. arenaria, B. xylophilus, Pratylenchus coffeae, Radopholus similis*, and *C. elegans* and the transcriptomes of *H. avenae, Longidorus elongatus, Rotylenchulus reniformis*, and *N. aberrans* using default parameters. A common set of 65 CEGs, previously defined as putatively single copy, was identified between all species tested. For each CEG, a protein alignment was carried out between all species using MUSCLE (Edgar 2004). The corresponding DNA sequence for each CEG in each species was back aligned using a custom python script (https://github.com/peterjc/pico_galaxy/tree/master/tools/align_back_trans, last accessed August 22, 2014). For each species, back alignments of CEGs or protein alignments were concatenated in the same order to generate super alignments. The super alignments were loaded into TOPALi (Milne et al. 2009) for model selection and maximum-likelihood tree generation using RaxML (WAG/GTR GAMMA) with 100 bootstraps. The phylogenetic tree was rerooted using *L. elongatus* as a known outgroup with FigTree v1.3.1 (http://tree.bio.ed.ac.uk/software/figtree/, last accessed August 22, 2014).

### Identification of Effectors

*Nacobbus* orthologues of “known effectors,” previously identified in other nematodes, were initially identified in the *N. aberrans* transcriptome using BLASTn with a minimum *e* value of 1e10^−^^15^ by default, followed by subsequent analysis of reciprocal BLAST hit, temporal expression profile and secretion signals. Signal peptides and transmembrane domains were predicted using SignalP v4.1 and TMHMM v2.0, respectively. Protein or DNA alignments were carried out using MUSCLE v3.8.3.1 (Edgar 2004). 4D06-like sequences were identified by BLAST as described above. Open reading frames were identified manually. Protein alignments were carried out as described above, and a phylogenetic analysis carried out in TOPAli using RaxML (WAG,GAMMA), midpoint rerooted, and formatted in Figtree v1.3.1. For identification of novel putative-effectors, assumptions were made about biologically relevant expression clusters, secreted proteins were predicted from these as above and compared with online databases using BLAST.

### Identification of Putative Nematode-Derived Plant Peptide Hormone Mimics

CLE-like or C-terminally encoded peptide-like (CEP) motifs were identified from the *N. aberrans* transcriptome using a pipeline similar to that previously described (Bobay et al. 2013). Protein-encoding regions of each transcript were predicted using Transdecoder with a minimum protein length of 50 amino acids. All peptides greater than 150 amino acids were discarded and the remaining were subject to signal peptide and transmembrane domain prediction as described above. Those that had a predicted signal peptide and lacked a transmembrane domain were queried for CLE-like or CEP-like domains using the permissive regular expressions [S|V|I]P[S|T|G]G[P|S][N|D]P and G[A-Z]S[A-Z]G[A-Z]GH, respectively. Sequences were then manually confirmed, and the relevant domains visualized using the MEME suite (Bailey et al. 2009). Due to the short size of these signaling peptides (12–13 amino acid), it was necessary to check that they were not present by chance alone. Therefore, each protein in the *N. aberrans* transcriptome was shuffled (thus maintaining amino acid representation), and the analysis described above was repeated.

## Results

### Transcriptome Assembly

In brief: 193 million reads across nine samples representing biological triplicates of three life stages were assembled into a single reference transcriptome containing 60,746 transcripts different enough to warrant independent analysis. CEGMA analysis showed that 96% of the CEGs were present as full length transcripts, with a further 1% represented by partial length transcripts. These data suggest that the majority of transcripts present in the transcriptome are likely to be full length. Postassembly contamination analysis identified 1,947 putative contaminants (primarily of fungal origin, supplementary fig. S1, Supplementary Material online) that were removed from subsequent analyses. The sequence reads have been submitted to SRA (accession numbers PRJEB6762). Further metrics are included in supplementary results section S1 and tables S1 and S2, Supplementary Material online.

### Lateral Gene Transfers

A total of 570 *N. aberrans* proteins returned an AI >30 and less than 70% identity to a nonmetazoan protein (supplementary table S3, Supplementary Material online). The corresponding genes were considered as acquired via LGT and unlikely to represent contamination (Gladyshev et al. 2008). Validating our approach, several previously reported and phylogenetically confirmed cases of LGT acquisitions in PPN were retrieved in this set of proteins. For example, GH5 cellulases, GH30 xylanases, GH28 polygalacturonases, GH32 candidate invertases, PL3 pectate lyases, L-threonine aldolases, candidate phosphoribosyltransferases, GH43 candidate arabinanases, GH53 candidate arabinogalactanase, chorismate mutases, expansin-like proteins, and Vitamin B5 pantothenate, all previously reported in different Clade 12 PPN (de Almeida Engler et al. 1999; Haegeman, Jones, et al. 2011) were identified. Interestingly, classes of enzymes, such as GH28 polygalacturonases, observed in root-knot nematodes (Jaubert et al. 2002) and their close relatives in the family Pratylenchidae (Haegeman, Joseph, et al. 2011; Bauters et al. 2014), but not in cyst nematodes, were identified in *N. aberrans*. Conversely, other classes of enzymes, such as GH53 candidate arabinogalactanase, reported in cyst nematodes (Vanholme et al. 2009) and suggested to be present in Pratylenchidae (Haegeman, Joseph, et al. 2011) but not in root-knot nematodes, were also found in *N. aberrans* (table 1). Transcripts encoding candidate glycoside hydrolase (GH) enzymes putatively acquired by LGT that are *N. aberrans* specific were also identified.

**Table 1:** Between Species Comparison of Laterally Acquired Genes

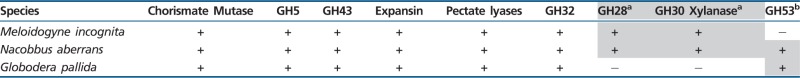

^a^Absent from cyst nematodes.

^b^Absent from root-knot nematodes.

Highlighted classes of interest.

### Whole Transcriptome Comparisons

A total of 3,262 different protein domains were found in 15,674 *N. aberrans* predicted proteins. This is within the range of domain diversity observed for the other nematodes (2,784 min–3,638 max) possibly indicative of a largely “complete” transcriptome (supplementary fig. S2, Supplementary Material online). Comparing *N. aberrans* to root-knot nematodes, cyst nematodes and *C. elegans* identified 2,444 conserved protein domains present in all the nematodes analyzed here. As many as 51 protein domains were specific to PPN. This includes several of the candidate LGT genes (as described above). We identified 40 protein domains shared between *N. aberrans* and the root-knot nematodes but not present in the cyst nematode *G. pallida* and conversely 31 protein domains shared between *N. aberrans* and *G. pallida* but not present in RKN genomes. In both cases root-knot nematode “specific” and cyst nematode “specific” laterally acquired genes were present in the *N. aberrans* transcriptome (as described above). A total of 134 protein domains were specific to *N. aberrans* with respect to cyst nematodes, root-knot nematodes, and *C. elegans*, 55 of which returned an AI greater than 30 and are therefore putative *N. aberrans*-specific LGT events.

### Differentially Expressed Transcript Clusters

**Fig. 2.—:**
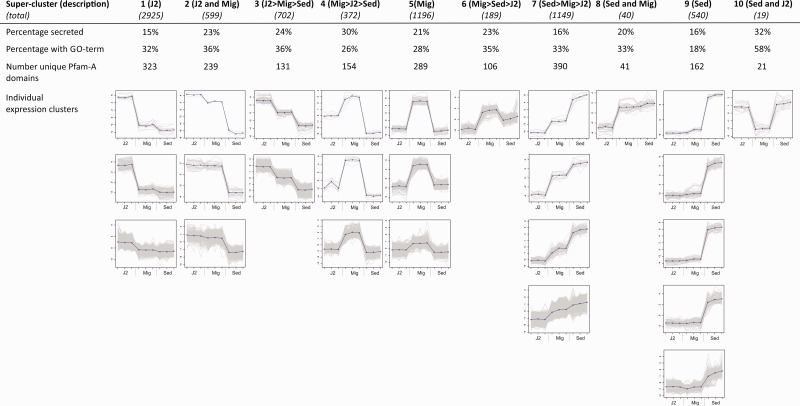
Transcripts clustered by differential expression. For all expression clusters, mean centred log fold change of expression is plotted for each of the three biological replicates for each life stage in the following order: J2, mixed J3/J4/female migratory (Mig), and sedentary female (Sed). A total of 7,731 differentially expressed transcripts clustered into 26 clusters based on expression profile. Clusters are manually grouped into ten super clusters with summarized expression.

Comparing normalized expression values between samples identified 7,731 transcripts that were differentially expressed with a minimum fold-change of 4 (*P* < 0.001). These could be grouped into 21 clusters that were manually assigned to ten super clusters (fig. 2). In general expression profiles of the differentially expressed transcripts are consistent between each of the three biological replicates (fig. 2). J2 specific (super cluster 1) was the most numerically dominant with almost 3,000 transcripts. On average, the majority (66%) of differentially expressed transcripts in all clusters had no assignable GO terms (fig. 2). Of the characterizable transcripts present in the J2 super cluster, a significant enrichment of GO terms associated with chemosensory behaviour, ion channel activity and proteins located at the neuronal cell body was observed using Fisher’s exact test (FDR 0.05). No significantly enriched GO terms were identified in the sedentary cluster (super cluster 9) using false discovery rate (FDR 0.05). However, using a minimum *P* value of 0.05, transcripts involved in regulation of vulval development and regulation of nematode larval development were overrepresented. The “migratory and sedentary” super cluster contained only 40 transcripts, 14 of which had associated GO terms. Two of these corresponded to membrane transport of sugars and were significantly over represented (FDR 0.05). Further analysis of super-clusters and conserved domains therein are covered in supplementary section S2 and table S4, Supplementary Material online.

### Multigene Phylogenetic History of Sedentary PPN

**Fig. 3.—:**
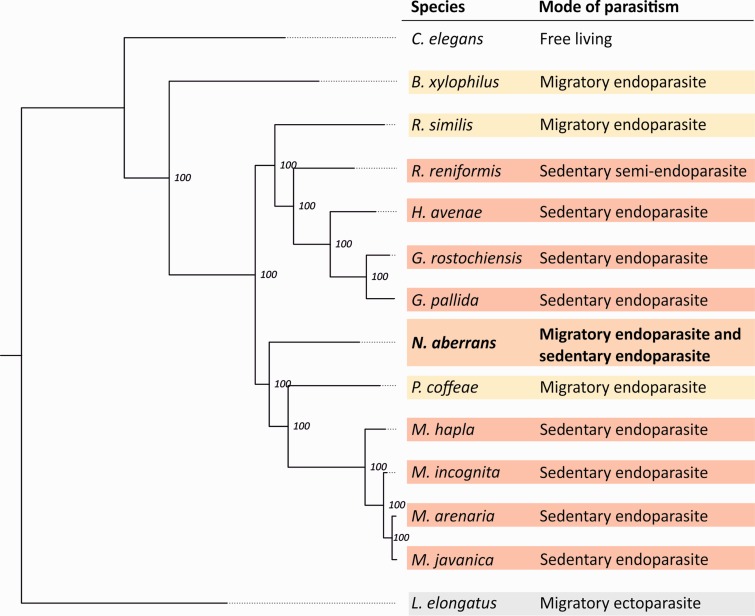
Local phylogenetic history of sedentary PPN: Nuclear protein-coding multigene phylogeny. Concatenated multigene protein alignment of 65 CEGs present in all 15 species. Using *Longidorus elongatus* as a root: *Nacobbus aberrans* lies at the base of the root-knot nematodes. Node numbers represent bootstrap support values for 100 iterations, and species are highlighted by their different life strategies.

We were able to identify 65 putatively single copy core eukaryotic genes (CEGs) from genome and transcriptome resources available for 15 species representing ten genera of the most economically important PPN related to *N. aberrans*. Using all species, with either a concatenated protein alignment or a codon aware nucleotide alignment of all 65 genes, we constructed a robust phylogeny representing the likely relationships between the major plant parasitic nematode clades. The topology described is in agreement with phylogenetic trees based on a single SSU rDNA sequence (Holterman et al. 2006, 2009; van Megen et al. 2009). Both our multigene phylogenetic analysis and SSU rDNA phylogenies performed so far place *N. aberrans* at the closest outgroup position relative to the root-knot nematodes and the *Pratylenchus* species (fig. 3). However, it is important to note that despite this apparent congruence, the phylogenetic position of *N. aberrans* fluctuates as a function of the set of species included in the phylogeny. For instance, removal of *P. coffeae* from the analysis causes *N. aberrans* to group with the cyst nematode clade; however, the additional removal of *R. similis* causes *N. aberrans* to group with the root-knot nematode clade for protein alignment but with the cyst nematode clade for back-threaded nucleotide alignment. With the presence/absence of the species described above, or with protein/codon aware nucleotide alignment, the relative positions of all other nematodes do not change in any case tested, only *N. aberrans* has different positions at the base of either the cyst nematode clade or the root-knot/pratylenchus nematode clade. The uncertainty in the positioning of *N. aberrans* may reflect its proximity to the bifurcation. Paradoxically, in any of the scenarios above, the position of *N. aberrans* was supported by strong (>70) boot strap support values.

### Comparing the *N. aberrans* Effector Complement to Other PPN

Due to the unusual biology of *N. aberrans* with similarities to both root-knot and cyst nematodes, the transcriptome was analyzed for the presence of effectors previously characterized from *M. incognita* (Huang et al. 2003) and *G. pallida* (Cotton et al. 2014). With the exception of the cell wall degrading and modifying proteins described above, very little overlap of the *M. incognita* effector proteins was seen with those of *N. aberrans* other than the few that were also present in *G. pallida.* This included the small signaling peptides discussed in detail below and a putatively secreted chorismate mutase. By contrast, of the 36 families of *G. pallida* putative effectors identified (Cotton et al. 2014), 16 were present in the *N. aberrans* transcriptome, the majority of which (12) contained at least one member with a predicted signal peptide. In general, expression patterns were consistent between the *G. pallida* effector families and the corresponding *N. aberrans* sequences (supplementary section S3 and table S5, Supplementary Material online). Taken together these results suggest functional homology for cyst nematode and *N. aberrans* effectors.

### Common Effectors between Root-Knot, Cyst and False Root-Knot Nematodes: Plant Peptide Mimics

**Fig. 4.—:**
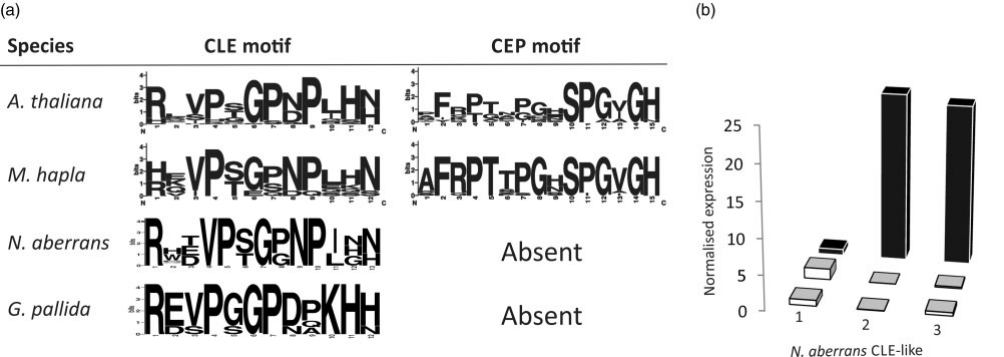
Comparison of nematode-derived plant peptide mimics between genera. (*a*) A comparison between the CLE and CEP-like peptides in *Arabidopsis thaliana* and PPN. No CEP-like sequences were identifiable in either *N. aberrans* or *Globodera pallida*. (*b*) Two of the three sequences containing CLE-like motifs in the *Nacobbus aberrans* transcriptome contain putative signal peptides (2 and 3) and have expression peaks during the sedentary life stage (2 and 3) compared with juvenile or migratory stages. Data for *A. thaliana* and *M. hapla* are modified from Bird et al. (Bird et al. 2014).

Both the root-knot and cyst nematodes secrete peptides into their host plant that have high sequence similarity to endogenous plant peptide-hormones. These short genes are up regulated at feeding stages and encode a predicted secretion signal, followed by a highly conserved 12–13 amino acid domain. To date, two classes of peptides have been characterized in nematodes, the CLE-like (or CLAVATA-like) and CEP-like (Gao et al. 2003; Wang et al. 2005, 2011; Lu et al. 2009; Bobay et al. 2013). Nine CLE-like sequences have been identified in root-knot nematode genomes and three were identifiable from the *G. pallida* genome (Cotton et al. 2014; Bobay et al. 2013). Three CLE-like peptides were also identifiable in the *N. aberrans* transcriptome, two of which contained putative signal peptides, and were significantly up regulated during the sedentary biotrophic phase (fig. 4). Conversely, CEP-like peptides have only been described from the root-knot nematode and are absent from *G. pallida*. No CEP-like peptides were identified in the *N. aberrans* transcriptome. As a control, no CLE-like or CEP-like domains were identified downstream of signal peptides in either a randomized transcriptome or proteome of *N. aberrans*.

### Common Effectors between Cyst Nematodes and False Root-Knot Nematodes: Putative Effector 4D06

**Fig. 5.—:**
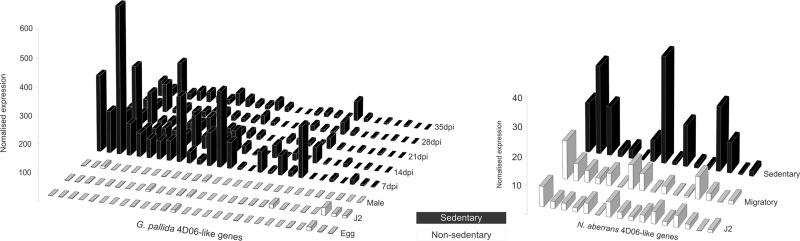
Expression comparison of 4D06-like gene families of *Globodera pallida* and *Nacobbus aberrans*. Each line contains expression data from a single gene across the life cycle. *G. pallida* 4D06-like genes are characterized by highest expression in the earliest stages of sedentary endoparasitism. *Nacobbus aberrans* 4D06-like sequences follow a similar expression pattern with peaks of expression during sedentary parasitic stages.

A family of effectors (4D06) was first identified from *H. glycines* (Gao et al. 2003). A large family of similar sequences is present in *G. rostochiensis* (Eves-van den Akker S, Unpublished data), *G. pallida* (Qin et al. 2000; Cotton et al. 2014), *R. reniformis* (Eves-van den Akker S, Unpublished data) and the limited information for *H. glycines* suggests that this nematode too harbours a large family of related proteins*.* Several 4D06-like sequences are present as a gene family in *N. aberrans*. Neither the cyst nematode nor the *N. aberrans* 4D06 sequences identified any significant hits from the genomes of the root-knot nematodes *M. incognita* or *M. hapla* by BLASTp (>30% identity across >40% of query length). Both *G. pallida* and *N. aberrans* 4D06-like genes encode putatively secreted proteins that are up regulated during the sedentary phase of the life cycle (fig. 5). These data further support a proposed function in syncytia but are in contrast to the proposed phylogenetic proximity of *N. aberrans* with root-knot nematodes.

**Fig. 6.—:**
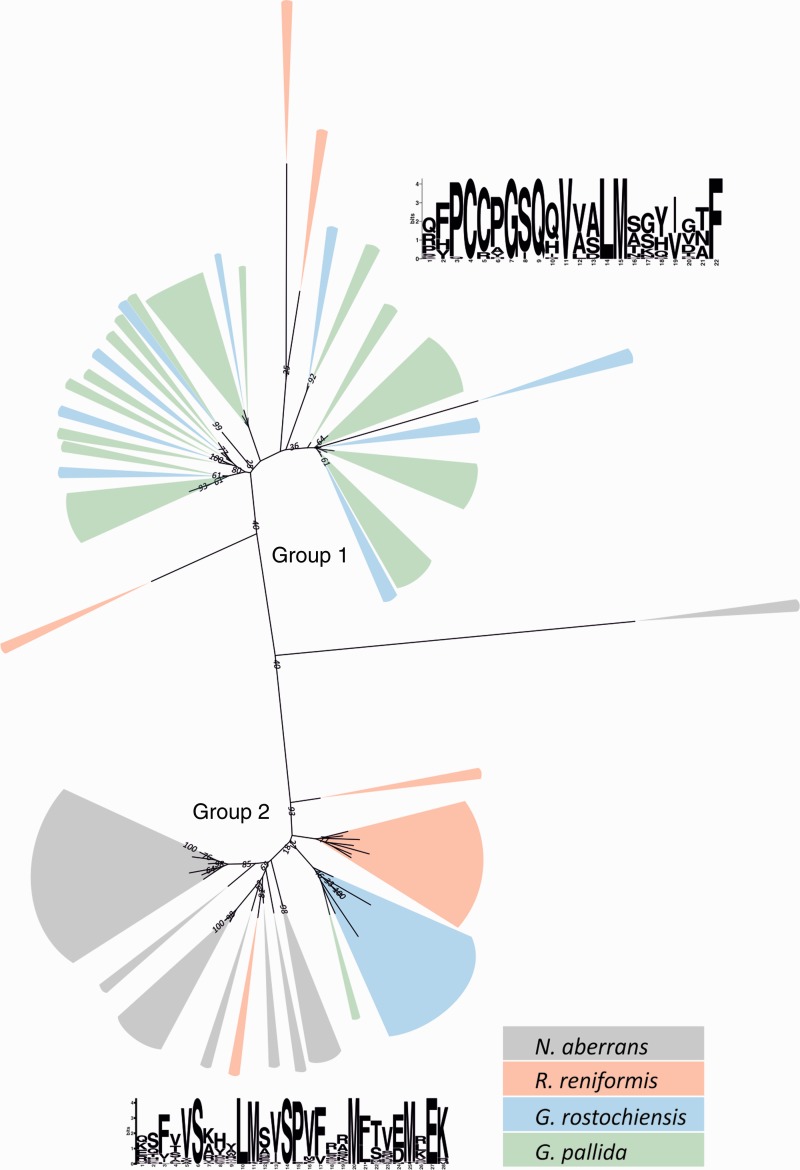
Putative effector 4D06: Multispecies protein alignment. Midpoint rooted protein phylogeny of 4D06-like sequences of *Nacobbus aberrans* (gray), *Rotylenchulus reniformis* (red), *Globodera pallida* (green), and *G. rostochiensis* (blue) is divided into two groups. Group 1 is dominated by cyst nematode sequences. Group 2 contains almost all of the *N. aberrans* sequences, 75% of *R. reniformis* sequences, 40% of *G. rostochiensis* sequences, and 3.4% of *G. pallida* sequences and is therefore presumably ancestral. MEME analysis has identified a highly conserved motif, termed “PCCP” that is present with a conserved location in all sequences in group 1 and absent in group 2.

4D06-like sequences were present in *N. aberrans* and all sedentary nematode species in the cyst nematode clade (including *R. reniformis*—fig. 3). However, a conserved domain present in most cyst nematode 4D06-like sequences was absent from the *N. aberrans* sequences (fig. 6). If the proposed phylogeny of PPN is to be believed, 4D06-like sequences appear to have been lost in *Meloidogyne* spp., the conserved domain appears to originate after the split from *N. aberrans*, and the expansion of sequences containing the conserved domain appears after the split from *R. reniformis*.

## Discussion

We present the first deep-sequencing whole transcriptome resource available for any false root-knot nematode species. We have generated a comprehensive database of genes expressed across three life stages, and have used robust statistical analyses to quantify changes in gene expression that occur in the transition between modes of parasitism. This analysis has revealed insights into the evolution, phylogenetic history, and biology of biotrophic plant-nematode interactions.

### Comparative Geno/Transcriptomics

Next generation sequencing, and in particular RNAseq, is an extremely powerful technique but the tools developed for analysis of the data that are generated are in their infancy. Most analyses, including phylogenomics, become more challenging with large numbers of isoforms. A pipeline was used to reduce redundancy while retaining as much “information” as possible using the most recent accepted methodology from a range of sources (Schliesky et al. 2012; Yang and Smith 2013). All nonredundant, nonchimeric transcripts were therefore treated independently, and the term gene is used tentatively. The final transcriptome assembly contained >60,000 transcripts less than 97% similar at the nucleotide level. This is many more than expected or observed in other PPN and is probably reflective of sequencing nematodes originally collected from a mixed soil sample and the presence of several isoforms that could not be collapsed into single genes. Similar numbers of common Pfam domains were identified when comparing *N. aberrans* with the root-knot nematode *M. incognita* and the cyst nematode *G. pallida*. In this analysis *N. aberrans* was more similar to either *M. incognita* or *G. pallida* than they are to each other, supporting the position at the base of the cyst nematode—root-knot nematode bifurcation.

### Lateral Gene Transfer

Hundreds of transcripts with alien indices indicative of laterally acquired genes were identified in the *N. aberrans* transcriptome. Several of these have been characterized in other nematode species as virulence factors with functioning GH activity on plant oligo- and polysaccharides (Jaubert et al. 2002; Haegeman, Jones, et al. 2011). Interestingly, classes of GH that are present in either root-knot nematodes or cyst nematodes are both present in *N. aberrans*. Of particular interest is the *N. aberrans* orthologue of the *M. incognita* protein Minc18543b. This has been identified as a functioning GH28 polygalacturonase and is expressed in the subventral gland cells (Jaubert et al. 2002). Until now GH28s were hypothesized to be root-knot nematode specific and possibly shared with their close Pratylenchidae relatives (Haegeman, Joseph, et al. 2011). However, two similar transcripts with high sequence identity to the *M. incognita* sequence have been identified in *N. aberrans*. The *N. aberrans* sequences are expressed during the J2, or both the J2 and sedentary life stages, and both contain putative signal peptides suggesting functional homology. Neither the *N. aberrans* nor the *M. incognita* GH28s identify any similar sequences in *G. rostochiensis* or *G. pallida.* A highly similar GH28 sequence was also identified from *R. reniformis*. However, the *R. reniformis* sequence appears to be nonfunctional as it lacks a signal peptide and has low read coverage during either J2 or sedentary phase (Eves-van den Akker S, unpublished data). Taken together these results may suggest that a common ancestor to root-knot and cyst nematodes had acquired GH28 polygalacturonase via LGT, and that a gradual loss of function has occurred during speciation in the cyst nematode clade. Interestingly, *N. aberrans*-specific candidate GH enzymes were also identified, although these remain to be confirmed and functionally characterized.

### Comparing Characterized Effector Complements between PPN

Putative plant peptide-hormone mimics are some of the best characterized effectors in PPN. CLE-like peptides have been identified in both cyst and root-knot nematodes and, unsurprisingly, were identifiable in the *N. aberrans* transcriptome. The CLE-like peptides identified were downstream of predicted secretion signals and were specifically expressed during the biotrophic phase in *N. aberrans*. The function of the CLE-like peptides in PPNs is not clear. CLEs are one of the few features that are shared between both cyst and root-knot nematodes leading to a suggestion that they play a fundamental role in the development of two morphologically diverse feeding sites. By contrast, CEP-like peptides have only been identified in root-knot nematodes and vascular plants (Bobay et al. 2013). CEP-like peptides were not identifiable in the *N. aberrans* transcriptome. According to the phylogenetic position of *N. aberrans*, this suggests that evolution/acquisition occurred after the split from *N. aberrans*. Despite their proposed function in root nodule formation (Imin et al. 2013), and the phenotypic similarities between nodules and galls, this suggests either a role unrelated to gall formation for these peptides or that superficially morphologically similar galls are produced by distinct means in different PPNs.

Of the 36 effector families identified in *G. pallida*, half were represented by at least one member in the *N. aberrans* transcriptome. The majority of these effectors are highly up regulated at J2 and have unknown functions. It is assumed that “effectors” expressed in the J2 stage of *G. pallida* play a role in either migration, suppression of host defences or initiation of the feeding site. Due to the unique biology of *N. aberrans*, by comparing these orthologues we can support or reject some of these hypotheses. It is reasonable to assume that genes involved in initiation of the feeding site are expressed in stages just prior to feeding site formation. In *G. pallida* this would correspond to the J2. However, all J2 specific effectors of *G. pallida* that have orthologues in *N. aberrans* are also J2 specific in that species. For *N. aberrans* the stage immediately prior to feeding site initiation would be an immature female, which is the developmental stage after the J3/4. This therefore suggests none of the J2 specific effectors shared by *G. pallida* and *N. aberrans* are involved in the initiation of the feeding sites, and that the as yet unidentified genes in either species, are strictly transcriptionally regulated. Those genes in *G. pallida* that are J2 and male specific are hypothesized to be involved in either migration or suppression of host defences. The corresponding orthologues in *N. aberrans* are up regulated at J2 and migratory stages. However, the migratory stage of *N. aberrans* causes necrosis and lesions, suggesting that these *G. pallida* genes are not involved in suppression of host defences, and are probably involved in migration. Finally, those genes where corresponding orthologues are expressed during the feeding stages in both species may play a critical and conserved role in maintenance of the feeding site or suppression of host defences.

One such gene family, similar to the 4D06 effector of *H. glycines* (Gao et al. 2003) was identified in cyst nematodes, reniform nematodes, and in *N. aberrans.* This is again in contrast with the proposed phylogeny (as for the other effectors shared by cyst nematodes and *Nacobbus*) and therefore suggests that a common ancestor of cyst and root-knot nematodes would have contained the 4D06 gene family. In addition, this gene family provides interesting insights into the evolution of sedentary endoparasitism. This gene family is present in at least three genera of PPN capable of infecting both Monocotyledons and Dicotyledons, suggesting a fundamental role in plant parasitism. A protein alignment of all 4D06-like effectors identified to date allows the sequences to be divided into two groups (fig. 6). Group 2, presumably the most “ancestral”, contains 100% of *N. aberrans*, 75% of *R. reniformis*, 40% of *G. rostochiensis*, and just 3.4% of *G. pallida* 4D06-like sequences. Protein alignments are often more reflective of function than evolutionary history, but coupled with the proposed phylogeny (fig. 3), this suggests that as speciation events have occurred, a gradual change from group 2 to group 1 4D06-like genes has taken place, and that they have been lost in the root-knot nematodes. Cyst nematodes, reniform nematodes, and *N. aberrans* are able to infect *Solanum tuberosum* with varying degrees of success. Both *N. aberrans* and *R. reniformis* have broad host ranges, whereas the potato cyst nematodes *G. pallida* and *G. rostochiensis* are specialists. However, 4D06-like genes have been identified in other cyst nematodes that do not infect potato (*H. avenae* ERP004648), suggesting that the switch to, and expansion of, group 1 4D06-like genes does not reflect adaptation toward a particular host.

Significantly differentially expressed transcripts were grouped into ten super clusters that described the changes in gene expression across the *N. aberrans* life cycle. As well as life stage specific clusters that have obvious biological relevance, we have identified clusters that describe transitions from juvenile to parasitic migratory endoparasite to parasitic sedentary endoparasite. Interestingly, the sedentary-specific super cluster 9 is the least well described with just 18% of transcripts with BLAST2GO hits. Making the reasonable assumption that genes specifically expressed at certain life stages are crucial to those stages, this highlights how little is known about this complex biotrophic interaction. Hundreds of putative effectors have been identified that have specific expression profiles, presumably separating the different functions required for successful parasitism of both migratory parasitic and sedentary parasitic stages. Effectors specifically up regulated during the J2 stage are predicted to be involved in migration, whereas those in the sedentary stage are likely to be involved in maintenance of the feeding site and suppression of host defences. Typical effector finding pipelines identify genes encoding secreted proteins specifically up regulated during the biotrophic (or migratory) phase of the interaction. This relatively crude analysis can identify a range of proteins that are not effectors (e.g., digestive enzymes). However, in the case of *N. aberrans,* the presence of a migratory stage that reportedly feeds but does not form biotrophic feeding sites gives more confidence in this type of analysis. Using a combination of known effector expression profiles and clustering of *N. aberrans* transcripts based on expression profile we can identify super clusters that contain putative effector sequences for further study.

### Evolution of Plant-Parasitism by Nematodes

Due to morphometric and physiological variability of *Nacobbus* in response to host range and temperature, there is very little agreement on the species taxonomy within the *N. aberrans* complex (Lax et al. 2013). Using the wealth of information generated here, a local phylogenetic history of the false root-knot nematodes is proposed. We present the most comprehensive and robust phylogeny of PPN, including representatives from all major groups, based on multiple nuclear protein-encoding genes. It is important to note that when constructing the phylogeny the relative positions of all species, except *N. aberrans,* remained constant with the addition of more species. *N. aberrans* was always positioned with the other Clade 12 nematodes, but was found in closer proximity to the root-knot nematodes or the cyst nematodes depending on the range of species included in the analysis. The 65 genes used here are highly conserved, present in all eukaryotes, predicted to be single copy and therefore are expected to be good indicators of evolutionary history. Despite the large number of conserved genes used here, it is possible that *N. aberrans* represents a rogue taxon and this may indicate insufficient phylogenetic signal (Sanderson and Shaffer 2002). The Pratylenchidae appear to be difficult to accurately position in a phylogenetic tree based on SSU rDNA, as *R. similis* groups with the cyst nematodes, whereas *N. aberrans* and the other Pratylenchinae group with the root-knot nematodes. Similarly, it has been noted that *B. xylophilus*, and indeed all Parasitaphelenchidae, are difficult to position using SSU rDNA due to unusual GC content (Holterman et al. 2006). However, for the 65 genes used here, no major differences were noted in GC content of the *B. xylophilus* nuclear genes compared with the other nematode species.

Using all common CEGs between all species available the proposed phylogenetic placement of *N. aberrans* is in agreement with published phylogenies. The phylogenetic proximity of *N. aberrans* to the root-knot nematodes correlates poorly with the biology of this nematode. With the exception of the galls, all other defining features of the biotrophic interaction are more similar to those of cyst and reniform nematodes than those of root-knot nematodes. For example, the juveniles of cyst nematodes migrate destructively and intracellularly (Wyss and Grundler 1992), whereas the juveniles of root-knot nematodes migrate intercellularly (Wyss and Grundler 1992; Atkinson et al. 1995). Like the cyst nematodes, the juveniles of *N. aberrans* also migrate destructively and intracellularly (Manzanilla-López et al. 2002). The syncytial feeding structure of *Nacobbus* is similar in structure and ontogeny to that of cyst nematodes, and in particular reniform nematodes (Jones and Payne 1977; Vovlas et al. 2007), and does not resemble the giant cells of root-knot nematodes. Females of root-knot nematodes, reniform nematodes, and false root-knot nematodes all produce eggs into a gelatinous matrix. In root-knot nematodes this matrix originates from rectal glands, whereas in reniform nematodes it originates from vaginal glands (Robinson et al. 1998). Although the origin of the *N. aberrans* gelatinous matrix is not known (Geraert 1994; Manzanilla-López et al. 2002), *N. aberrans* and all sedentary nematodes in the cyst/reniform nematode clade lack rectal glands. Indeed, the biology of *N. aberrans* is remarkably similar to that of *R. reniformis*; both moult from J2 to J3/4 female in the soil, retaining their cuticle. The female is the infective stage (i.e., able to induce syncytia) in both and, in sub optimal conditions the J3/J4 is the survival stage.

These observations can be put in parallel with the LGT analysis and the effector complement analysis. Classes of GH enzyme present in either root-knot nematodes or cyst nematodes are both present in the *N. aberrans* transcriptome. In addition, cyst nematode specific effectors, absent from root-knot nematodes, are also present in the *N. aberrans* transcriptome. Similarly, the difficulties in positioning *N. aberrans* with respect to cyst and root-knot nematode clades depending on the number of species, number of sequences, or the type of alignment used (as discussed above) may indicate that it is at the very base of this bifurcation. Varying reports exist in the literature about the evolutionary origins of sedentary endoparasitism in the phylum Nematoda (Holterman et al. 2009; van Megen et al. 2009; de Almeida Engler and Gheysen 2013). The position of *R. similis* and *P. coffeae* on each side of the bifurcation presumably implies that migratory endoparasitism is a prerequisite to sedentary endoparasitism and that the migratory parasitic stages of sedentary endoparasites have been lost in cyst and root-knot nematodes and yet retained as an important part of the life cycle in the “intermediate” genus *Nacobbus*.

The wealth and quality of the information here has raised interesting questions into the phylogeny of PPN and provides a tremendous resource for further study into both sedentary and migratory parasitism. As the only known nematode to have both migratory and sedentary phases of the life cycle, *N. aberrans* had provided valuable information, and may become a useful model system, for both fields.

## Supplementary Material

Supplementary sections S1–S5, tables S1–S5, and figures S1 and S2 are available at *Genome Biology and Evolution* online (http://www.gbe.oxfordjournals.org/).

Supplementary Data
