# Supplementary material for: The Transcriptome of Nacobbus aberrans Reveals Insights into the Evolution of Sedentary Endoparasitism in Plant-Parasitic Nematodes
Source: Genome Biol Evol. 2014 Aug 13;6(9):2181–94. doi: 10.1093/gbe/evu171 (PMC4202313; doi:10.1093/gbe/evu171)
Supplement: Supplementary Data [file supp_6_9_2181__index.html]

The transcriptome of Nacobbus aberrans reveals insights into the evolution of sedentary endoparasitism in plant-parasitic nematodes — The Transcriptome of Nacobbus aberrans Reveals Insights into the Evolution of Sedentary Endoparasitism in Plant-Parasitic Nematodes — Supplementary Data 

# The Transcriptome of *Nacobbus aberrans* Reveals Insights into the Evolution of Sedentary Endoparasitism in Plant-Parasitic Nematodes

## Supplementary Data

files

**Files in this Data Supplement:**

- Supplementary Data - pdf file
- Supplementary Data - docx file
- Supplementary Data - xlsx file
- Supplementary Data - xlsx file
- Supplementary Data - xlsx file
- Supplementary Data - xlsx file
