# Supplementary material for: The Transcriptome of Nacobbus aberrans Reveals Insights into the Evolution of Sedentary Endoparasitism in Plant-Parasitic Nematodes
Source: Genome Biol Evol. 2014 Aug 13;6(9):2181–94. doi: 10.1093/gbe/evu171 (PMC4202313; doi:10.1093/gbe/evu171)
Supplement: Supplementary Data [file supp_evu171_TableS4.pdf]

Super-cluster (description)	1 (J2)	2 (J2 and Mig)	3 (J2>Mig>Sed)	4 (Mig>J2>Sed)	5 (Mig)	6 (Mig>Sed>J2)	7 (Sed>Mig>J2)	8 (Sed and Mig)	9 (Sed)	10 (Sed and J2)
Pfam-A domains	2TM	2Fe-2S_thioredx	2OG-FeII_Oxy	Abhydrolase_3	2OG-FeII_Oxy	3Beta_HSD	14-3-3	2OG-FeII_Oxy	3Beta_HSD	7TM_GPCR_Srsx
	7tm_1	7tm_1	4_1_CTD	Activin_recp	3Beta_HSD	7tm_3	2OG-FeII_Oxy	3HCDH	7tm_1	AhpC-TSA
	7tm_2	7tm_3	7tm_1	Acyl_transf_3	7tm_1	Aa_trans	2OG-FeII_Oxy_2	3HCDH_N	7tm_7	Aldolase_II
	7tm_3	7TM_GPCR_Srsx	7tm_2	adh_short	7tm_3	Abhydrolase_5	3Beta_HSD	7TM_GPCR_Srsx	7TM_GPCR_Sra	ANF_receptor
	7tm_7	7TM_GPCR_Sru	7tm_3	AhpC-TSA	7TM_GPCR_Sra	Abhydrolase_6	3HCDH	AAA_5	7TM_GPCR_Srab	Arf
	7TM_GPCR_Sra	7TM_GPCR_Srv	7TM_GPCR_Srd	Aldolase_II	7TM_GPCR_Srab	Amidase_2	3HCDH_N	Aldolase_II	7TM_GPCR_Srd	Astacin
	7TM_GPCR_Srab	7TM_GPCR_Srw	7TM_GPCR_Srsx	ALO	7TM_GPCR_Srb	AMP-binding	7tm_1	Ank	7TM_GPCR_Srh	CAP
	7TM_GPCR_Srb	7TM_GPCR_Srx	7TM_GPCR_Srt	Ank	7TM_GPCR_Srsx	AMP-binding_C	7TM_GPCR_Srab	Ank_2	7TM_GPCR_Sri	Catalase
	7TM_GPCR_Srbc	AAA	7TM_GPCR_Srw	Ank_2	7TM_GPCR_Srv	An_peroxidase	7TM_GPCR_Srsx	Ank_3	7TM_GPCR_Srt	Catalase-rel
	7TM_GPCR_Srd	AAA_17	7TM_GPCR_Srx	Ank_3	7TM_GPCR_Srw	Asp	7TM_GPCR_Srt	Ank_4	7TM_GPCR_Sru	CUB
	7TM_GPCR_Srh	AA_permease	7TM_GPCR_Str	Ank_4	7TM_GPCR_Srx	Astacin	7TM_GPCR_Srv	Asp	7TM_GPCR_Srv	DAGK_cat
	7TM_GPCR_Sri	AA_permease_2	AA_permease	Ank_5	AAA_22	Bestrophin	AAA	Calponin	7TM_GPCR_Srw	DUF4071
	7TM_GPCR_Srj	Aa_trans	Abhydrolase_3	An_peroxidase	AA_permease	CAP	AAA_11	CH	7TM_GPCR_Srx	FumaraseC_C
	7TM_GPCR_Srsx	ABC_ATPase	Abhydrolase_6	ASC	AA_permease_2	cEGF	AAA_12	Col_cuticle_N	7TM_GPCR_Str	G-alpha
	7TM_GPCR_Srt	ABC_membrane	Acyltransferase	Asn_synthase	Aa_trans	COesterase	AAA_16	Collagen	AAA	Glutaredoxin
	7TM_GPCR_Sru	ABC_tran	adh_short	Asp	Abhydrolase_2	Col_cuticle_N	AAA_17	dsrm	AAA_12	Glyco_hydro_25
	7TM_GPCR_Srv	Abhydrolase_3	adh_short_C2	Asp_protease_2	Abhydrolase_3	Collagen	AAA_18	EamA	AAA_2	Glyco_hydro_28
	7TM_GPCR_Srw	Actin	AhpC-TSA	Astacin	Abhydrolase_5	CUB	AAA_19	ELO	AAA_5	Guanylate_cyc
	7TM_GPCR_Srx	Acyl-CoA_dh_1	Aminotran_1_2	BACK	Abhydrolase_6	Cyclin_C	AAA_2	EmrE	Aa_trans	Lyase_1
	7TM_GPCR_Str	Acyl-CoA_dh_2	AMP-binding	BTB	Actin	Cyclin_N	AAA_22	F420_oxidored	Abhydrolase_3	Pectate_lyase_3
	A1_Propeptide	Acyl-CoA_dh_M	AMP-binding_C	bZIP_Maf	Acyl-CoA_dh_1	DAO	AAA_25	FA_desaturase	Acetyltransf_1	Peptidase_M1
	AAA	Acyl-CoA_dh_N	ANF_receptor	C2-set_2	Acyl-CoA_dh_2	DB	AAA_30	F-box	Acyl-CoA_dh_1	
	AAA_11	Acyl_transf_1	Ank	C4	Acyl-CoA_dh_M	DCX	AAA_33	Glycos_transf_2	Acyl-CoA_dh_2	
	AAA_12	Acyl_transf_3	Ank_2	Cadherin	Acyl-CoA_dh_N	DLH	AAA_5	Glyco_tranf_2_2	Acyl-CoA_dh_M	
	AAA_17	adh_short	Ank_3	Ca_hom_mod	Acyltransferase	DM13	Aa_trans	Glyco_tranf_2_3	Acyltransferase	
	AAA_2	Amidohydro_1	Ank_4	Calponin	ADAM_spacer1	DOCK-C2	ABC_membrane	Glyco_transf_7C	adh_short	
	AAA_21	Amidohydro_5	Ank_5	CBFD_NFYB_HMF	adh_short	DUF1986	ABC_tran	Glyco_transf_7N	AhpC-TSA	
	AAA_28	Amnionless	Annexin	CBM_2	AhpC-TSA	DUF236	Abhydrolase_1	HA2	AICARFT_IMPCHas	
	AAA_30	AMP-binding	APOBEC_N	cEGF	Amidohydro_5	DUF290	Abhydrolase_3	Helicase_C	ALO	
	AAA_34	ANF_receptor	Apr	Chitin_synth_2	Amnionless	DUF4139	Abhydrolase_6	Kinase-like	Ammonium_transp	
	AAA_5	Ank	Arf	CoA_transf_3	AMOP	DUF4140	Abhydro_lipase	Kinesin	Ank	
	AAA_6	Ank_2	Arrestin_C	COesterase	AMP-binding	EamA	ACBP	MCM	Ank_2	
	AAA_7	Ank_3	Arrestin_N	Col_cuticle_N	Ank	EB	Acetyltransf_1	MCM_N	Ank_3	
	AAA_8	Ank_4	ASC	Collagen	Ank_2	EF-hand_2	Acetyltransf_10	Meth_synt_1	Ank_4	
	AAA_9	Ank_5	Asp	CRAL_TRIO	Ank_3	EF-hand_3	Acetyltransf_13	Meth_synt_2	Ank_5	
	AAA-ATPase_like	An_peroxidase	Asp_Arg_Hydrox	Ctr	Ank_4	EF-hand_5	Acetyltransf_4	Mg_chelatase	ASC	
	AA_permease	Arf	Astacin	CUB	Ank_5	EF-hand_6	Actin	Neur_chan_LBD	Astacin	
	AA_permease_2	ASC	B56	Cyt-b5	An_peroxidase	EF-hand_7	Acyl-CoA_dh_1	Nuc_sug_transp	BTB	
	Aa_trans	Asp	Band_7	DB	Arf	EF-hand_8	Acyl-CoA_dh_2	OB_NTP_bind	CAP	
	ABC_ATPase	Asparaginase_2	Bestrophin	DERM	ARF7EP_C	EGF_3	Acyl-CoA_dh_M	P4Ha_N	Carb_anhydrase	
	ABC_membrane	Astacin	Bin3	DOMON	ARID	EGF_CA	Acyl-CoA_dh_N	PCI	Catalase	
	ABC_tran	ATP-gua_Ptrans	BTB_2	DPBB_1	ASC	ELO	Acyl_transf_3		Catalase-rel	
	Abhydrolase_3	ATP-gua_PtransN	C1_1	DSPc	Asn_synthase	Endonuclea_NS_2	Acyltransferase		CBM_14	
	Abhydrolase_6	Band_3_cyto	C2	DUF1647	Asp	Epimerase	ADAM_spacer1		Cellulase	
	Acetyltransf_1	Band_7	Cache_1	DUF1794	Astacin	ERAP1_C	ADH_N		Claudin_2	
	Acetyltransf_7	Bestrophin	Calponin	DUF2650	B56	FKBP_C	adh_short		CLN3	
	Activin_recp	Biopterin_H	Calsequestrin	DUF316	BACK	Fork_head	adh_short_C2		ClpB_D2-small	
	Acyl-CoA_dh_1	BiPBP_C	Calx-beta	DUF4139	Band_7	GCS	ADH_zinc_N		CMAS	
	Acyl-CoA_dh_2	BK_channel_a	CAP	DUF4140	Bestrophin	GCV_T	ADK		COesterase	
	Acyl-CoA_dh_M	Branch	Carb_anhydrase	DUF4499	Beta-lactamase	GCV_T_C	ADK_lid		Col_cuticle_N	
	Acyl-CoA_dh_N	BTB_2	CarboxypepD_reg	DUF791	BK_channel_a	Globin	AhpC-TSA		Collagen	

Super-cluster (description)	1 (J2)	2 (J2 and Mig)	3 (J2>Mig>Sed)	4 (Mig>J2>Sed)	5 (Mig)	6 (Mig>Sed>J2)	7 (Sed>Mig>J2)	8 (Sed and Mig)	9 (Sed)	10 (Sed and J2)
	Acyltransferase	bZIP_1	Carn_acyltransf	EB	BTB	Glyco_hydro_2	AIG1		CPG4	
	Adaptin_N	bZIP_2	Cation_ATPase_C	EF-hand_1	BTB_2	Glyco_hydro_2_C	AIG2		Crust_neurohorm	
	ADH_N	bZIP_Maf	Cation_ATPase_N	EF-hand_5	bZIP_1	Glyco_hydro_2_N	Aldedh		CUB	
	adh_short	C1_1	CBM_2	EF-hand_6	bZIP_2	Glyco_transf_11	Aldolase_II		DAGAT	
	adh_short_C2	C2	CBM49	EF-hand_7	bZIP_Maf	Ground-like	Aldose_epim		DB	
	ADH_zinc_N	Ca_chan_IQ	Cellulase	EF-hand_8	C4	hEGF	ALO		DEAD	
	ADH_zinc_N_2	Cache_1	CH	EGF	Cadherin	Hexokinase_1	Amidase		DIX	
	ADK	Cadherin	Claudin_3	EGF_3	Calx-beta	Hexokinase_2	Amidinotransf		DUF1647	
	AdoMet_MTase	Calponin	Clc-like	EGF_CA	CAP_N	Hint	Amidohydro_5		DUF1679	
	AhpC-TSA	CAP	cNMP_binding	ELO	Catalase	Histone	Amino_oxidase		DUF1943	
	AICARFT_IMPCHas	CH	COesterase	EMI	Catalase-rel	Hormone_recep	Aminotran_1_2		DUF229	
	Aldedh	Claudin_2	Copine	Ephrin	Cation_ATPase_C	HSP20	Aminotran_4		DUF258	
	Aldo_ket_red	Clc-like	Crust_neurohorm	Exo_endo_phos	Cation_ATPase_N	ICL	Aminotran_5		DUF290	
	Aldolase_II	CLP_protease	Cu2_monoox_C	FAD_binding_4	Cauli_VI	IQ	AMOP		DUF4071	
	Amidinotransf	cNMP_binding	Cu2_monooxygen	FA_hydroxylase	CBS	Kinase-like	AMP-binding		DUF750	
	Aminotran_1_2	COesterase	CUE	F-box	CFEM	Kunitz_BPTI	AMP-binding_C		DUF953	
	Ammonium_transp	Col_cuticle_N	CUT	F-box-like	Clc-like	LBP_BPI_CETP	Ank		Dynamin_M	
	Amnionless	Collagen	DB	FERM_M	CMAS	LBP_BPI_CETP_C	Ank_2		Dynamin_N	
	AMOP	Copine	DPBB_1	FERM_N	COesterase	Lectin_C	Ank_3		EcKinase	
	AMP-binding	CPG4	DREV	FKBP_C	Col_cuticle_N	LIM	Ank_4		EF-hand_8	
	AMP-binding_C	CRAL_TRIO	DTW	Frag1	Collagen	LRR_1	Ank_5		ELO	
	ANF_receptor	Crust_neurohorm	DUF1448	Fringe	CP2	LRR_4	An_peroxidase		Epimerase	
	Ank	CUB	DUF148	FumaraseC_C	CRAL_TRIO	LRR_5	APH		EST1	
	Ank_2	Cu-oxidase_3	DUF2650	FXa_inhibition	Crust_neurohorm	LRR_8	Arch_ATPase		EST1_DNA_bind	
	Ank_3	DB	DUF290	Gal-bind_lectin	CTD_bind	Lustrin_cystein	Arf		FAD_binding_4	
	Ank_4	DEP	DUF3250	GATA	CUB	Malate_synthase	Arm		F-box	
	Ank_5	DKCLD	DUF3399	GATase_6	Cytochrom_B561	MARVEL	Asp		Folate_carrier	
	APH	DUF1136	DUF3694	GATase_7	DB	Metallophos	Asp_protease_2		Fork_head	
	Apr	DUF148	DUF4440	GCS	DEAD	Methyltransf_22	Astacin		Galactosyl_T	
	Arf	DUF1647	Dynamin_M	GILT	Destabilase	MMPL	AT_hook		Gal-bind_lectin	
	ARID	DUF1679	Dynamin_N	Glyco_hydro_19	Dimer_Tnp_hAT	Motile_Sperm	ATP-cone		GATase_2	
	Arrestin_C	DUF1794	Dynein_light	Glycoprotein_B	DnaJ	Moulting_cycle	BIR		GATase_4	
	Arrestin_N	DUF1899	E1-E2_ATPase	Glyco_tran_28_C	DNA_pol_B_2	MTHFR	Branch		GATase_6	
	ASC	DUF1900	EF-hand_1	Glyco_tranf_2_3	DND1_DSRM	MtN3_slv	Bromodomain		GATase_7	
	Asp	DUF1943	EF-hand_4	Glyco_trans_1_3	DOCK-C2	Myosin_head	BTB		GBP	
	Asp_protease	DUF2424	EF-hand_5	Glyco_trans_2_3	DOMON	MyTH4	BTB_2		GDC-P	
	Asp_protease_2	DUF3381	EF-hand_6	Glyco_transf_11	DPBB_1	NAD_binding_10	BTG		Globin	
	Astacin	Dynein_light	EF-hand_7	Glyco_transf_92	DSPc	NAD_binding_4	BtpA		Glyco_hydro_25	
	ATP-synt_ab	EB	EF-hand_8	Ground-like	dsrm	NmrA	bZIP_1		Glyco_hydro_2_C	
	ATP-synt_ab_C	EFG_C	EGF_CA	GSHPx	DUF1258	p450	bZIP_2		Glyco_hydro_30	
	ATP-synt_ab_N	EF-hand_1	EMP70	GSH_synthase	DUF1295	PAN_1	C1_1		Glyco_hydro_31	
	B9-C2	EF-hand_2	FA	GSH_synth_ATP	DUF1768	PAN_3	Calponin		Glyco_hydro_47	
	Baculo_RING	EF-hand_3	FAD_binding_1	GST_C	DUF1943	PAP2	Calreticulin		Glyco_tran_28_C	
	Band_7	EF-hand_5	FARP	GST_N	DUF2424	Patched	CAMSAP_CH		GTP_EFTU	
	BBS1	EF-hand_6	FERM_C	GST_N_3	DUF273	PDZ	CAP		GTP_EFTU_D2	
	Beta-Casp	EF-hand_7	FERM_M	hEGF	DUF290	PDZ_2	Catalase		GTP_EFTU_D3	
	Biopterin_H	EF-hand_8	FERM_N	Hint	DUF316	PEP_mutase	Catalase-rel		Guanylate_cyc	
	Branch	EGF	FHA	His_Phos_2	DUF423	Pepsin-I3	Cation_efflux		GWT1	
	Bromodomain	EGF_3	Flavodoxin_1	Histone	DUF4360	Peptidase_A22B	CBFD_NFYB_HMF		HATPase_c	
	BTB	EGF_CA	FLYWCH	Homeobox	DUF4582	Peptidase_M1	CBM_14		HATPase_c_3	
	BTB_2	Endonuclease_NS	fn3	Hormone_recep	DUF750	Peptidase_M13	CDT1		HEAT_2	

Super-cluster (description)	1 (J2)	2 (J2 and Mig)	3 (J2>Mig>Sed)	4 (Mig>J2>Sed)	5 (Mig)	6 (Mig>Sed>J2)	7 (Sed>Mig>J2)	8 (Sed and Mig)	9 (Sed)	10 (Sed and J2)
	BTD	Epimerase	FYVE_2	HSP20	Dynamin_M	Peptidase_M13_N	cEGF		Helicase_C	
	bZIP_1	ERAP1_C	Gal-bind_lectin	HTH_23	Dynamin_N	Peptidase_M14	CFEM		Hint	
	bZIP_2	EST1_DNA_bind	GBP	HTH_28	E1-E2_ATPase	Peptidase_MA_2	CH		Hint_2	
	bZIP_Maf	Exo_endo_phos	GDE_C	ig	EB	Peptidase_S9	Choline_kinase		His_Phos_1	
	C1_1	EXOSC1	GED	Ig_2	EFF-AFF		Chromo		His_Phos_2	
	C1_3	FA	Gelsolin	Ig_3	EF-hand_1		CLASP_N		Histone	
	C2	F-box	Globin	Ion_trans_2	EF-hand_5		Clat_adaptor_s		HNOBA	
	C6	F-box-like	Glutaminase	I-set	EF-hand_6		Claudin_2		Homeobox	
	Cache_1	FERM_C	Glyco_hydro_28	Kazal_2	EF-hand_7		CMAS		Homeobox_KN	
	Cadherin	FERM_M	Glyco_hydro_30	KR	EF-hand_8		COesterase		Hormone_recep	
	Calponin	FERM_N	Glyco_hydro_32N	Kunitz_BPTI	EGF		Col_cuticle_N		HSP20	
	Calreticulin	FHA	Glyco_tran_28_C	Laminin_EGF	EGF_3		Collagen		HSP70	
	Calx-beta	FLYWCH	Glyco_trans_1_3	Laminin_G_1	EGF_CA		COPIIcoated_ERV		HSP90	
	CaMBD	fn3	Glyco_transf_11	Laminin_G_2	ELO		Cpn10		IBR	
	CAMSAP_CH	Fork_head	Glyco_transf_7C	LBP_BPI_CETP	Endonuclea_NS_2		Cpn60_TCP1		Ig_2	
	CAP	FragX_IP	Glyco_transf_7N	LBP_BPI_CETP_C	Endonuclease_NS		CRAL_TRIO		Ig_3	
	CAP_GLY	FtsJ	GoLoco	Ldl_recept_a	Epimerase		Creatinase_N		Innexin	
	CAP_N	Gal-bind_lectin	Ground-like	Lectin_C	Epimerase_Csub		CSD		I-set	
	Carb_anhydrase	Gal_Lectin	GSHPx	LIM	ERG4_ERG24		CTD_bind		Kinesin	
	CarboxypepD_reg	G-alpha	GST_C	Lipase_2	Evr1_Alr		CUB		Kunitz_BPTI	
	Carn_acyltransf	GBP	GST_N	Lipase_3	Exo_endo_phos		Cullin		LBP_BPI_CETP	
	Cast	GCC2_GCC3	GTP_EFTU	LRR_1	Exo_endo_phos_2		Cullin_Nedd8		LBP_BPI_CETP_C	
	Cation_ATPase_C	G-gamma	Gtr1_RagA	LRR_7	FAA_hydrolase		Cyclin_C		Ldl_recept_a	
	Cation_ATPase_N	GHMP_kinases_N	HAD	LRR_8	FAD_binding_3		Cyclin_N		Lectin_C	
	Cauli_VI	Glyco_hydro_56	HEAT	Lyase_1	FGF		Cys_Met_Meta_PP		LicD	
	CBS	Glycos_transf_2	Helicase_C	Macin	FLYWCH		Cyt-b5		Lipase_2	
	CD20	Glyco_tran_28_C	HLH	MBOAT	FMO-like		DAG1		Lipase_3	
	CDC48_2	Glyco_tranf_2_2	Homeobox	Metallophos	fn3		DB		LRR_1	
	cEGF	Glyco_tranf_2_3	Homeobox_KN	Methyltransf_21	Fork_head		dCMP_cyt_deam_1		LRR_4	
	Cellulase	Glyco_trans_1_3		Methyltransf_26	Frag1		DC_STAMP		LRR_8	
	CH	Glyco_transf_10		MFS_1	Frizzled		DDOST_48kD		MaoC_dehydratas	
	Chromo	Glyco_transf_7C		Moulting_cycle	FumaraseC_C		DEAD		MaoC_dehydrat_N	
	Claudin_2	GNAT_acetyltr_2		MRF_C1	Galactosyl_T		DEP		Methyltransf_11	
	Cluap1	GPCR_chapero_1		MRF_C2	Gal-bind_lectin		Destabilase		Methyltransf_15	
	CM_2	Gp-FAR-1		Myosin_head	Gamma-thionin		DIOX_N		Methyltransf_18	
	CN_hydrolase	Ground-like		MyTH4	GATase_6		DKCLD		Methyltransf_23	
	cNMP_binding	GTP_EFTU		NAD_synthase	GATase_7		DnaJ		Methyltransf_25	
	CNRIP1	GTP_EFTU_D2		NDT80_PhoG	GBP		DPM3		Methyltransf_31	
	Coatomer_WDAD	GTP_EFTU_D3		NNMT_PNMT_TEMT	GCC2_GCC3		dsrm		MFS_1	
	COesterase	Gtr1_RagA		Orai-1	GED		DTW		MMR_HSR1	
	Col_cuticle_N	HA2		PAN_1	Globin		DUF1074		MreB_Mbl	
	Collagen	HCO3_cotransp		Patched	Glucosamine_iso		DUF1253		MutS_II	
	COP-gamma_platf	HEAT		PAX	Glyco_hydro_18		DUF1387		MutS_III	
	CPSF73-100_C	HEAT_2		Pectate_lyase	Glyco_hydro_19		DUF1759		MutS_V	
	CRAL_TRIO	HLH		Pep_M12B_propep	Glyco_hydro_25		DUF1768		NAD_binding_10	
	Crust_neurohorm	Homeobox		Peptidase_C1	Glycos_transf_1		DUF1899		NAD_binding_4	
	Ctf8	Hormone_recep		Peptidase_M10	Glycos_transf_2		DUF1900		NAD-GH	
	CUB	HSL_N		Peptidase_M14	Glyco_tran_28_C		DUF1917		Neur_chan_LBD	
	Cullin	HSP20		Peptidase_S10	Glyco_tranf_2_2		DUF2075		Neur_chan_memb	
	Cullin_Nedd8	ig		Peptidase_S74	Glyco_tranf_2_3		DUF229		NTR	
	CX	Ig_2		Peptidase_S9	Glyco_trans_4_4		DUF236		Orn_Arg_deC_N	

Super-cluster (description)	1 (J2)	2 (J2 and Mig)	3 (J2>Mig>Sed)	4 (Mig>J2>Sed)	5 (Mig)	6 (Mig>Sed>J2)	7 (Sed>Mig>J2)	8 (Sed and Mig)	9 (Sed)	10 (Sed and J2)
	Cyt-b5	Ig_3		Peptidase_S9_N	Glyco_transf_4		DUF273		Orn_DAP_Arg_deC	
	DAGK_acc	Innexin			Glyco_transf_7C		DUF290		p450	
	DAGK_cat	Ins145_P3_rec			Glyco_transf_92		DUF2974		PARP	
	DAG_kinase_N	Ins_beta			G-patch		DUF316		Patched	
	DAO	Integrin_beta			Ground-like		DUF3585		PAZ	
	dCMP_cyt_deam_1	Ion_trans			GSH_synthase		DUF3591		PDZ	
	DcpS_C	Ion_trans_2			GSH_synth_ATP		DUF4507		Peptidase_C1	
	DCX	IPPT			GTP_EFTU		DUF750		Peptidase_C1_2	
	dDENN	I-set			GTP_EFTU_D2		DUF758		Peptidase_C97	
	DDE_Tnp_IS1595	Iso_dh			Guanylate_kin		Dynamin_M			
	DEAD	Kdo			HA2		Dynamin_N			
	DegT_DnrJ_EryC1	Kelch_1			HAD		Dynein_light			
	DENN	Kelch_3			hEGF		E1_dh			
	DEP	Kelch_4			Helicase_C		E1-E2_ATPase			
	Det1	Kelch_5			Hint		EamA			
	DHC_N1	Kelch_6			HLH		EF-hand_1			
	DHC_N2	ketoacyl-synt			HlyIII		EF-hand_5			
	DHO_dh	Ketoacyl-synt_C			HMG_box		EF-hand_6			
	DM	KH_1			HMG_box_2		EF-hand_7			
	DnaJ	KH_3			Homeobox_KN		EF-hand_8			
	DNA_pol_B_2	Kinase-like			Hormone_recep		EGF			
	DNase_II	Kinesin			HSCB_C		EGF_CA			
	DREV	KR			HSP20		ELMO_CED12			
	DSHCT	Kunitz_BPTI			HtrL_YibB		ELO			
	DSPc	Laminin_G_1			Hydrolase		EmrE			
	dsrm	Laminin_G_2			Hydrolase_like2		EphA2_TM			
	DUF1041	LBP_BPI_CETP_C			HYR		Ephrin_lbd			
	DUF1042	Lectin_C			IBR		Epimerase			
	DUF1280	LepA_C			Ig_2		ERGIC_N			
	DUF1448	Lig_chan			Ig_3		ER_lumen_recept			
	DUF1647	Lig_chan-Glu_bd			Inhibitor_I29		Evr1_Alr			
	DUF1679	LIM			Innexin		Exo_endo_phos			
	DUF1736	Linker_histone			Ion_trans		F420_oxidored			
	DUF1758	LRAT			Ion_trans_2		F5_F8_type_C			
	DUF1759	LRR_1			IPK		FAD_binding_3			
	DUF1768	LRR_4			IRF-2BP1_2		FAD_binding_4			
	DUF1794	LRR_5			I-set		FA_desaturase			
	DUF1899	LRR_6			Kdo		FAD-oxidase_C			
	DUF1900	LRR_7			Kinase-like		F-box			
	DUF1981	LRR_8			K_oxygenase		F-box-like			
	DUF2045	Ly-6_related			Ku		FDF			
	DUF229	malic			Ku_C		FeoB_N			
	DUF2371	Malic_M			Ku_N		Ferritin			
	DUF2464	Melibiase			Kunitz_BPTI		FGGY_C			
	DUF273	Methyltransf_11			L27_1		FGGY_N			
	DUF290	Methyltransf_12			La		FHA			
	DUF3381	Methyltransf_18			Lactamase_B		Filamin			
	DUF3399	Methyltransf_23			Laminin_B		FLYWCH			
	DUF4098	Methyltransf_25			Laminin_EGF		FMO-like			
	DUF4480	Methyltransf_31			Laminin_G_1		Folate_carrier			
	DUF4507	MFS_1			Laminin_G_2		Formyl_trans_C			

Super-cluster (description)	1 (J2)	2 (J2 and Mig)	3 (J2>Mig>Sed)	4 (Mig>J2>Sed)	5 (Mig)	6 (Mig>Sed>J2)	7 (Sed>Mig>J2)	8 (Sed and Mig)	9 (Sed)	10 (Sed and J2)
	DUF547	MgtE			Laminin_G_3		Formyl_trans_N			
	DUF605	MH1			Laminin_II		FR47			
	DUF788	MH2			Laminin_N		Fringe			
	DuoxA	MIP			LBP_BPI_CETP		Furin-like			
	Dus	MIR			LBP_BPI_CETP_C		FYVE			
	Dynamin_M	Miro			Ldl_recept_a		G2F			
	Dynamin_N	MORN			Ldl_recept_b		G6PD_C			
	Dynein_heavy	Motile_Sperm			Lectin_C		G6PD_N			
	Dynein_light	Moulting_cycle			LIM		Gal-bind_lectin			
	Dzip-like_N	MtN3_slv			Lipase_2		Gal_mutarotas_2			
	E1-E2_ATPase	MULE			Lipase_3		G-alpha			
	EB	Myosin_head			LRR_1		Gamma-thionin			
	EcKinase	Myosin_N			LRR_4		GBP			
	EF1G	Myosin_tail_1			LRR_5		GBP_C			
	EFG_C	Na_H_Exchanger			LRR_6		GED			
	EFG_II	Na_sulph_symp			LRR_7		GF_recep_IV			
	EFG_IV	NCD3G			LRR_8		Glyco_hydro_18			
	EF-hand_1	Nebulin			Lustrin_cystein		Glyco_hydro_25			
	EF-hand_4	Neur_chan_LBD			Lyase_1		Glyco_hydro_2_N			
	EF-hand_5	Neur_chan_memb			MADF_DNA_bdg		Glyco_hydro_30			
	EF-hand_6	NHL			Methyltransf_11		Glyco_hydro_31			
	EF-hand_7	NPR2			Methyltransf_21		Glyco_hydro_32N			
	EF-hand_8	NRDE-2			Methyltransf_22		Glyco_hydro_43			
	EFhand_Ca_insen	Nup160			Methyltransf_23		Glyco_hydro_47			
	EF-hand_like	OB_NTP_bind			Methyltransf_25		Glycoprotein_B			
	EGF	OLF			Methyltransf_31		Glycos_transf_1			
	EGF_CA	p450			MFP2b		Glycos_transf_2			
	eIF2A	PAZ			MFS_1		Glyco_tranf_2_2			
	ELFV_dehydrog	PBP			MFS_1_like		Glyco_tranf_2_3			
	ELFV_dehydrog_N	PDZ			MFS_2		Glyco_trans_1_4			
	EMP70	Peptidase_C1			MGAT2		Glyco_transf_54			
	Endonuclea_NS_2	Peptidase_M1			Miro		Glyco_transf_7C			
	Endonuclease_NS	Peptidase_M13			MMPL		Glyco_transf_7N			
	Epimerase	Peptidase_M13_N			MMR_HSR1		Glyco_transf_92			
	Esterase_phd	Peptidase_MA_2			Mob1_phocein		Glypican			
	Ets				Motile_Sperm		GON			
	Exo_endo_phos				Moulting_cycle		Granulin			
	Exo_endo_phos_2				MTHFR		GRASP55_65			
	FA				MtN3_slv		GST_C			
	FAD_binding_4				MULE		HA2			
	FA_desaturase				Myb_DNA-bind_6		HATPase_c			
	FAD-oxidase_C				Na_Ca_ex		HATPase_c_3			
	FARP				NAD_binding_10		HDA2-3			
	F-box				NAD_binding_4		HECA			
	F-box-like				NAD_binding_8		hEGF			
	FERM_C				NAD_binding_9		Helicase_C			
	FERM_M				NAD_Gly3P_dh_C		Hexapep			
	FERM_N				NAD_Gly3P_dh_N		Hexapep_2			
	FHA				NAD_synthase		HHH_5			
	Fip1				Na_H_Exchanger		Hint			
	FLYWCH				NCD2		Hint_2			

Super-cluster (description)	1 (J2)	2 (J2 and Mig)	3 (J2>Mig>Sed)	4 (Mig>J2>Sed)	5 (Mig)	6 (Mig>Sed>J2)	7 (Sed>Mig>J2)	8 (Sed and Mig)	9 (Sed)	10 (Sed and J2)
	FMO-like				Neur_chan_LBD		His_Phos_1			
	fn3				Neur_chan_memb		His_Phos_2			
	Fork_head				NHL		Histone			
	Frizzled				NIDO		HLH			
	FtsJ				NTP_transf_2		HMG_box			
	FYVE_2				Nucleotid_trans		HMG_box_2			
	Fzo_mitofusin				OB_NTP_bind		Homeobox			
	GAF				p450		Homeobox_KN			
	GAF_2				PAM2		HORMA			
	GAF_3				PAN_1		Hormone_recep			
	gag-asp_proteas				PAP2		HSCB_C			
	Galactosyl_T				PAP2_C		HSP20			
	Gal-bind_lectin				PAP_central		HSP70			
	G-alpha				PAP_RNA-bind		HSP90			
	GBP				PAS		HTH_23			
	GCV_T				PAS_11		HTH_28			
	GCV_T_C				Patatin		IBB			
	GED				Patched		IBR			
	GFRP				PAX		IF4E			
	G-gamma				PAZ		ig			
	Gln-synt_C				PDZ		Ig_2			
	Gln-synt_N				PDZ_2		Ig_3			
	Globin				Pectate_lyase		Inhibitor_I29			
	Glutaminase				Pepsin-I3		Innexin			
	Glyco_hydro_18				Peptidase_C1		Ion_trans			
	Glyco_hydro_19				Peptidase_C1_2		Ion_trans_2			
	Glyco_hydro_20				Peptidase_C97		IPPT			
	Glyco_hydro_30				Peptidase_M1		IPT			
	Glyco_hydro_56				Peptidase_M14		IP_trans			
	Glyco_tran_28_C				Peptidase_M2		IQ			
	Glyco_trans_1_3				Peptidase_M8		I-set			
	Glyco_transf_10				Peptidase_S10		Isochorismatase			
	Glyco_transf_11				Peptidase_S28		IU_nuc_hydro			
	Glyco_transf_43				PET		KAP_NTPase			
	Glypican						Kazal_1			
	GMP_PDE_delta						Kazal_2			
	GNAT_acetyltr_2						Kdo			
	GpcrRhopsn4						KH_1			
	Ground-like						KH_3			
	GSHPx						Kinase-like			
	GST_C						Kinesin			
	GST_C_3						KR			
	GST_N						Laminin_B			
	GTP_EFTU						Laminin_EGF			
	GTP_EFTU_D2						Laminin_N			
	GTP_EFTU_D3						Ldl_recept_a			
	Gtr1_RagA						Ldl_recept_b			
	Guanylate_cyc						Lectin_C			
	Guanylate_kin						Leuk-A4-hydro_C			
	HAD						LicD			
	HATPase_c						Lipase_3			

Super-cluster (description)	1 (J2)	2 (J2 and Mig)	3 (J2>Mig>Sed)	4 (Mig>J2>Sed)	5 (Mig)	6 (Mig>Sed>J2)	7 (Sed>Mig>J2)	8 (Sed and Mig)	9 (Sed)	10 (Sed and J2)
	HATPase_c_3						Lipocalin			
	HbrB						Lipocalin_7			
	HECT						LRR_1			
	hEGF						LRR_4			
	Helicase_C						LRR_5			
	Helicase_C_4						LRR_7			
	Helicase_RecD						LRR_8			
	His_Phos_1						LSM			
	Hist_deacetyl						LSM14			
	HIT						LuxC			
	HLH						Ly-6_related			
	HMG_box						Mac			
	HMG_box_2						Mannosyl_trans2			
	HMG_CoA_synt_C						MAP65_ASE1			
	HNOBA						MBOAT			
	Homeobox						MCLC			
	Homeobox_KN						MCM			
							MCM2_N			
							MCM_N			
							Metallophos			
							Met_gamma_lyase			
							Methyltransf_11			
							Methyltransf_18			
							Methyltransf_23			
							Methyltransf_25			
							Methyltransf_31			
							MFS_1			
							MGAT2			
							Mg_chelatase			
							Mic1			
							MIF4G			
							MIG-14_Wnt-bd			
							Misat_Tub_SegII			
							Mito_carr			
							MMR_HSR1			
							MoCF_biosynth			
							Moulting_cycle			
							MreB_Mbl			
							MtN3_slv			
							Myelin_PLP			
							Myosin_head			
							Myosin_N			
							Myosin_tail_1			
							NACHT			
							NAD_binding_10			
							NAD_binding_2			
							NAD_binding_4			
							NAD_binding_8			
							NCD1			
							NCD2			
							Ndc1_Nup			

Super-cluster (description)	1 (J2)	2 (J2 and Mig)	3 (J2>Mig>Sed)	4 (Mig>J2>Sed)	5 (Mig)	6 (Mig>Sed>J2)	7 (Sed>Mig>J2)	8 (Sed and Mig)	9 (Sed)	10 (Sed and J2)
							Neur_chan_LBD			
							Neur_chan_memb			
							NIDO			
							NKAIN			
							NTP_transf_3			
							NTP_transferase			
							Nuc_sug_transp			
							OB_NTP_bind			
							ORC4_C			
							Orn_Arg_deC_N			
							Otopetrin			
							OTU			
							p450			
							P5CR_dimer			
							PA			
							PALP			
							PAP2_C			
							Patatin			
							Patched			
							PAX			
							PAZ			
							PBC			
							PCNA_C			
							PCNA_N			
							Pectate_lyase			
							Peptidase_C1			
							Peptidase_C1_2			
							Peptidase_M1			
							Peptidase_M10			
							Peptidase_M14			
							Peptidase_M20			
							Peptidase_M24			
							Peptidase_MA_2			
