# Supplementary material for: The Transcriptome of Nacobbus aberrans Reveals Insights into the Evolution of Sedentary Endoparasitism in Plant-Parasitic Nematodes
Source: Genome Biol Evol. 2014 Aug 13;6(9):2181–94. doi: 10.1093/gbe/evu171 (PMC4202313; doi:10.1093/gbe/evu171)
Supplement: Supplementary Data [file supp_evu171_Supplementary_data_final.docx]

**Supplementary results:**

**Section S1: Transcriptome assembly and contamination removal**

RNA extracted from three life stages of *N. aberrans* was sequenced, each in biological triplicate. A total of 193 million reads were generated, with an average of 21 million reads per sample (Summarised in Table S1). All reads were pooled and subsequently trimmed and assembled using Trinity into 168,000 transcripts and 53,056 components (loose Trinity proxy for genes). Seven percent (11,448) of the transcripts were identified as *trans*-chimeric, the majority of which when cut were <200 base pairs and so were discarded. Having trimmed chimeric transcripts, redundancy among all transcripts was reduced further. 7,894 additional transcripts could now be fully contained within other transcripts at 99% nucleotide identity. This gave a final transcriptome of 60,746 unique, non-chimeric transcripts different enough to warrant independent analysis (Table S2).

Open Reading Frames (ORFs) were predicted from the *N. aberrans* transcriptome. A total of 1,947 *N. aberrans* proteins returned an Alien Index (AI) >0 and ≥70 % identity at the protein level with non-metazoan proteins. They were considered as putative contaminants and discarded from the rest of the analysis. The vast majority of these putative contaminants (1,628) had high identity matches to Fungi, with various diverse *Fusarium spp* accounting for most of these contaminations. 129 putative contaminants returned matches with ≥70 % identity to *Solanum lycopersicum*, the second largest single source of putative contamination (Figure S1).

**Section S2: Identification of novel candidate effectors**

For identification of novel putative-effectors, assumptions were made about biologically relevant expression clusters. Robust differential expression analysis relies on multiple biological replicates, as used here, but also on the software used ([Anders and Huber 2010](#_ENREF_1); [Rapaport et al. 2013](#_ENREF_2); [Soneson and Delorenzi 2013](#_ENREF_3)). Approximately 12 % of the transcripts were identified as differentially expressed at greater than 4 fold (P < 0.001). In both the J2 specific cluster (1) and the sedentary specific cluster (9) 15 % and 20 % of the transcripts respectively encoded secreted proteins (Figure S2). As is common to many putative effectors, the majority of the secreted proteins in the sedentary or J2 specific clusters had no conserved Pfam domains. In addition, numerous putative effectors were identified with no significant sequences identified by BLASTp (evalue 0.000005) to the *M. incognita* genome or the *G. pallida* genome. Those with Pfam A domains in the sedentary cluster corresponded to various glycoside hydrolases (covered in the main text), and several domains associated with ligand/DNA binding (Table S4). Several proteins associated with sex determination were also identified in the secreted sedentary-specific super cluster (9) and are therefore probably not effectors.

**Section S3: Corresponding *G. pallida* and *N. aberrans* effectors**

Putative effectors that were constitutively expressed in all *G. pallida* life stages (GPLIN_000442900, GPLIN_000393900) had corresponding orthologues with expression in all sequenced stages of *N. aberrans* (Nab_25904_c0_seq1, Nab_30330_c0_seq1). Those that are J2 specific in *G. pallida* (GPLIN_000555600, GPLIN_000604400, GPLIN_000977100) had corresponding orthologues in *N. aberrans* expressed at J2 (Nab_59981_c0_seq1, Nab_59984_c0_seq1, Nab_22156_c0_seq1). Those that were primarily J2 and male specific in *G. pallida* (GPLIN_000169700, GPLIN_001475500) had corresponding putative orthologues in *N. aberrans* expressed at J2 and migratory stages (Nab_26770_c0_seq1, Nab_29306_c0_seq1). Finally, those that were feeding stage specific in *G. pallida* had putative orthologues that were feeding stage specific in *N. aberrans*, one of which (4D06-like) is discussed in more detail in the main text.

**Section S4: 4D06 phylogeny**

For full sequence names used to construct the 4D06 phylogeny please refer to the following tree representation:

((N.abberans_58074_c0_seq1|m.54455:7.3962910,(R.reniformis_44171_c1_seq2:0.6166040,(((R.reniformis_38382_c0_seq1:0.5795610,(R.reniformis_38897_c0_seq1:0.4128750,R.reniformis_45849_c5_seq8:0.7547020)39:0.0467440)22:0.1017160,((R.reniformis_45845_c3_seq11:0.5249090,R.reniformis_32671_c1_seq1:0.8772040)49:0.0997160,(R.reniformis_43560_c0_seq3:0.4157380,R.reniformis_34646_c0_seq1:0.4439640)30:0.2141370)14:0.1685500)77:0.4824190,((G.rostochiensis_46010_c1_seq4:0.4513870,((G.rostochiensis_49597_c2_seq2:1.1426360,(G.rostochiensis_47509_c1_seq16:0.3049780,G.rostochiensis_51635_c3_seq2:0.3088710)100:0.2519750)33:0.1352370,(G.rostochiensis_39134_c0_seq1:1.3812460,(G.rostochiensis_47509_c1_seq20:0.6218470,G.pallida_001431400:0.6059070)21:0.1170540)14:0.1700440)6:0.1361740)46:0.7174090,((N.abberans_58785_c0_seq1|m.56859:0.0905620,N.abberans_29810_c0_seq1|m.32554:0.1081000)98:0.8612870,((N.abberans_17746_c0_seq1|m.13388:0.9069080,((N.abberans_22102_c0_seq1|m.17747:0.3760310,R.reniformis_45351_c1_seq2:0.6783220)81:0.1585880,(N.abberans_28814_c0_seq1|m.30060:0.6511710,(N.abberans_25528_c0_seq1|m.23566:0.2869160,(N.abberans_58078_c0_seq1|m.54462:0.0318420,N.abberans_58074_c0_seq1|m.54454:0.0432280)100:0.2810050)98:0.7755550)39:0.0784740)62:0.1684610)39:0.1585690,(N.abberans_28825_c0_seq1|m.30097:0.7283770,((N.abberans_14506_c0_seq1|m.11515:0.4626620,(N.abberans_22345_c0_seq1|m.18110:0.3819860,N.abberans_22689_c0_seq1|m.18608:0.6159910)64:0.0699050)62:0.1294270,(N.abberans_25912_c0_seq1|m.24242:0.3122250,(N.abberans_1502_c0_seq1|m.3910:0.4698320,(N.abberans_54230_c0_seq1|m.43712:0.0000000,N.abberans_54229_c0_seq1|m.43711:0.0000000)100:0.3503080)76:0.1844520)50:0.0969770)98:0.5586110)85:0.2631040)25:0.0709140)65:0.5466420)18:0.1280160)27:0.6738090)93:3.0262520)40:1.5210730,(R.reniformis_45915_c0_seq13:2.3645960,((((G.pallida_001255700:0.0000000,(G.pallida_000860700:0.0832620,G.pallida_001162100:0.4728710)93:0.3415570)61:0.0539040,(G.pallida_000792900:0.2097970,(G.pallida_000481100:0.0489340,(G.rostochiensis_50257_c1_seq3:0.0000000,G.pallida_000203300:0.0000000)97:0.0526230)61:0.0279880)43:0.0377610)99:0.3492660,((G.pallida_001038900:0.2718850,(G.rostochiensis_51009_c2_seq6:0.0610340,G.pallida_000796500:0.3026780)100:0.1276440)94:0.0969390,(G.pallida_000060800:0.1461780,(G.rostochiensis_46020_c0_seq1:0.2493180,(G.pallida_001471200:0.1095000,G.pallida_000388900:0.3661670)77:0.0788770)58:0.0509700)80:0.1181590)85:0.2085790)80:0.1075260,((G.rostochiensis_45890_c0_seq1:0.0000000,G.pallida_001221800:0.0207730)99:0.9644290,((G.pallida_001606400:0.1248120,(G.pallida_000970100:0.2285120,(G.pallida_000970000:0.0160930,(G.pallida_000969900:0.3012220,(G.rostochiensis_50478_c0_seq7:0.0000000,G.pallida_000912100:0.0000000)93:0.1290000):0.0000010)4:0.0214660)25:0.0363690)99:0.7377730,((R.reniformis_12501_c0_seq1:4.4037440,R.reniformis_42077_c0_seq10:1.8163130)25:1.4877890,((G.rostochiensis_44762_c0_seq2:0.1654790,(G.pallida_000407400:0.0000000,G.pallida_001456900:0.0062230)92:0.0988260)100:1.2725840,(G.pallida_001582700:0.1278680,((G.pallida_000950100:0.0000000,G.pallida_000126500:0.3556800)64:0.0117020,((G.pallida_000950600:0.0398750,G.pallida_000243800:0.0555310)29:0.0074910,(G.rostochiensis_12493_c0_seq1:3.4570190,((G.rostochiensis_50464_c3_seq4:0.0961570,(G.pallida_001443600:0.3009110,G.pallida_000309000:0.1671960)29:0.0678340)5:0.0072930,(G.pallida_001390400:0.0613760,(G.pallida_000243700:0.0189920,G.rostochiensis_50464_c3_seq2:0.0470800)61:0.0966760)46:0.0658200)3:0.0025930)1:0.0108870)4:0.0161760)13:0.1451120)53:0.4437440)36:0.1186340)14:0.4547420)6:0.2294500)8:0.0856060)25:1.2551570)40:1.0008760)

**Section S5: Plant parasitic multi-gene phylogeny**

Phylogeny of plant-parasitic nematode tre file, re-rooted by *L. elongatus* as a known out-group to produce phylogeny in the main text.

(M.arenaria:0.0084731,(M.incognita:0.0077186,(M.hapla:0.0461041,(P.coffeae:0.2081633,(N.aberrans:0.2040904,((B.xylophilus:0.3384864,(L.elongatus:0.4651018,C.elegans:0.3637066)100:0.1016511)100:0.1946270,(R.similis:0.2477418,(R.reniformis:0.1370819,(H.avenea:0.1030251,(G.rostochiensis:0.0524713,G.pallida:0.0630509)100:0.0817825)100:0.0828962)100:0.0423117)100:0.0444211)100:0.0316677)100:0.0427081)100:0.1741167)100:0.0417746)100:0.0193178,M.javanica:0.0103543)

**Figure S1 Putative contaminant transcripts removed**. A total of 1947 putative contaminant were identified with an alien index ≥0 and a protein identity to non-metazoans ≥ 70 %. The majority of contaminants present are from various *Fusarium spp*. The most abundant plant contamination was from *Solanum lycopersicum*.


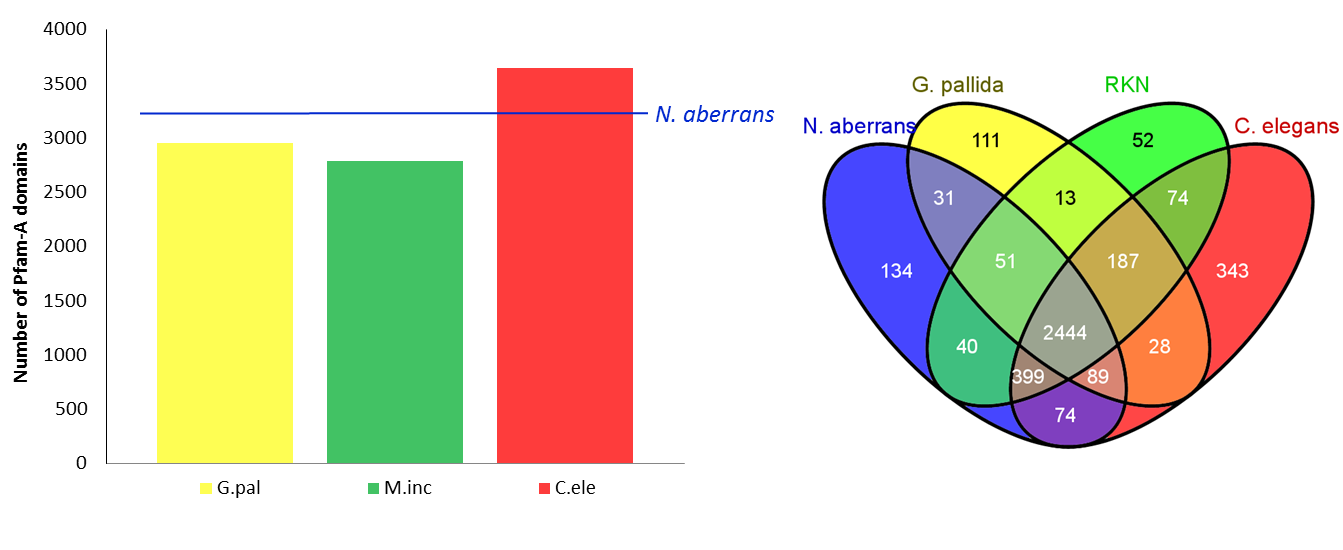
**Figure S2 Pfam diversity and comparison to published genomes.** Pfam –A domains were identified in all predicted proteins from the genomes of *G. pallida*, *M. incognita*, *C. elegans* and the transcriptome of *N. aberrans*. The diversity of Pfam domains in the *N. aberrans* transcriptome is within the range expected for complete genomes. Common Pfam domains between species are described in a Venn diagram.

**Supplementary references**

Anders S, Huber W. 2010. Differential expression analysis for sequence count data. Genome Biol 11.

Rapaport F, Khanin R, Liang Y, Pirun M, Krek A, Zumbo P, Mason CE, Socci ND, Betel D. 2013. Comprehensive evaluation of differential gene expression analysis methods for RNA-seq data. Genome Biol 14.

Soneson C, Delorenzi M. 2013. A comparison of methods for differential expression analysis of RNA-seq data. BMC Bioinformatics 14:1-18.
